# Strain, Chain, Repeat: Synthesis and Optoelectronic Properties of Poly(Naphthalene Benzene Vinylene)s

**DOI:** 10.1021/acsmacrolett.6c00194

**Published:** 2026-06-03

**Authors:** Bibek Dhungel, Mia Klopfenstein, Anahita Keer, Matthew D. Hannigan, Susanna Monroe, Jordan M. Garber, Wandy Rodriguez, Stephanie S. Lee, Marcus Weck

**Affiliations:** Molecular Design Institute, Department of Chemistry, 5894 New York University, New York, New York 10012, United States

## Abstract

Poly­(phenylenevinylene)­s (PPV)­s are a class of fluorescent conjugated polymers used in organic light emitting diodes (OLEDs). Living chain growth polymerization of PPVs is typically achieved by ring-opening metathesis polymerization (ROMP) of highly strained cyclophanedienes. While benzene-based cyclophanedienes are routinely reported, living or controlled ROMP of naphthalene-benzene containing mixed cyclophanedienes remains a challenge. This work describes the synthesis and polymerization of an alkoxy substituted naphthalene-benzene cyclophanediene using Schrock’s molybdenum olefin metathesis catalyst. We demonstrate the efficacy of this strategy in producing poly­(naphthalene benzene vinylene) (PNBV)­s with controlled molecular weights and narrow dispersities. The produced PNBVs are highly fluorescent polymers in solution and as thin films with distinct emission signatures arising from the benzene and naphthalene chromophores. An unoptimized PNBV thin film transistor exhibited a hole mobility of 8 × 10^–9^ cm^2^/V·s, which increased to 1 × 10^–7^ cm^2^/V·s upon doping with lithium bis­(trifluoromethanesulfonyl) imide (LiTFSI) and (tris­(2-(1*H*-pyrazol-1-yl)-4-*tert*-butylpyridine)­cobalt­(III) (FK 209). This expansion of ROMP to include the naphthalene moiety as well as a mixed-aromatic system will usher further development of electronically diverse PPVs with advanced solid-state applications.

## Graphical abstract

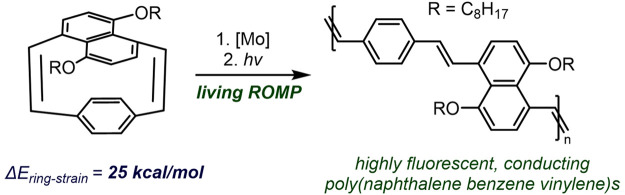

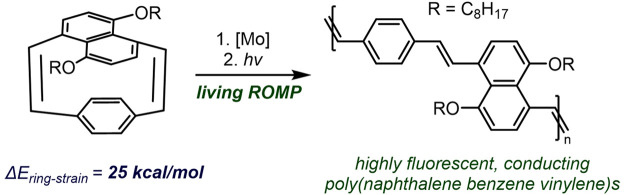

Fluorescent and conductive conjugated polymers are key components for organic light-emitting diodes (OLEDs),^1^ organic photovoltaics,^2^ organic field effect transistors,^3^ and more.^4–6^ Poly­(*p*-phenylenevinylene)­s or PPVs are among the most studied π-conjugated polymers and were famously incorporated into the first OLED in 1990.^7–9^ PPVs feature a distinct vinylic bond, which can be stereoselectively synthesized to remain in the *cis*–*trans* or *all-trans* conformation.^10–12^ *All-trans* PPVs are a relatively air and temperature stable polymer featuring increased planarity and enhanced effective conjugation length, making it an attractive candidate for use in optoelectronics.^13–16^ Common methods to synthesize PPVs include the Gilch, Wittig, Heck, Knoevenagel, and other step-growth methods,^17,18^ which offer little control over key polymer properties, such as molecular weights, dispersity, and end-groups, and limit the ability to form block copolymers, highlighting the need for a controlled polymerization method to access PPVs and analogous systems.^19^

Ring-opening metathesis polymerization (ROMP) – a chain growth polymerization method – has been previously shown to be effective in the living or highly controlled polymerization to yield PPVs (Scheme 1).^10,20–22^ Turner and co-workers first demonstrated living ROMP of a solution processable PPV using a using a tetra-alkoxy substituted [2.2] para-cyclophane-1,9-diene monomer.^23^ Cyclophanedienes carry significant strain energies resulting from their “bent and twisted” aromatic decks (10–20° dihedral twists) confined within the cyclic monomer structure (Figure 1).^10,24–26^ The release of the ring-strain favors living ROMP. Following the seminal report in 2006, Turner,^27–29^ Weck,^21,30–32^ Michaudel,^11–13^ Elacqua,^33^ and others reported living ROMP of similar benzene-based systems (Scheme 1a). ROMP of naphthalene containing cyclophanedienes is a logical extension to diversify the monomer scope. Another underdeveloped area is the incorporation of mixed-arene monomer systems resulting in alternating polymers. The incorporation of naphthalene units can influence the overall electronics of the final polymer and the presence of a mixed naphthalene-benzene system could potentially give rise to interesting optoelectronic properties that are influenced by alternating arene interactions between the two rings.^4–6,33–36^ Indeed, Yu and co-workers demonstrated the ROMP of a symmetrical naphthalene-based system primarily as a block copolymer with a benzene-based cyclophane diene (Scheme[Fig sch1]b).^37^ Our group reported the synthesis of a mixed naphthalene-benzene based cyclophanediene.^38^ Despite a ring strain of 25 kcal/mol, however, the naphthalene cyclophane diene monomer was found to be inactive to ROMP by both Grubbs’ and Schrock’s olefin metathesis catalysts (Scheme 1c).^38,39^ Structural information based on DFT calculations indicate increased steric bulk due to the substitution pattern around the dienes as the likely cause for inactivity. Interestingly, the system developed by Yu and co-workers has the same substitution pattern around arenes but is ROMP active, albeit at elevated temperatures (>90 °C), that is atypical in living or controlled ROMP. We attribute this to a likely lowered steric bulk around the dienes in their symmetrical system as opposed to the unsymmetrical naphthalene-benzene system reported earlier by us. Experimental or computed structural information, however, is not currently available to further comment on this hypothesis. To date, the living or highly controlled ROMP of a naphthalene-based symmetrical or mixed arene cyclophane diene system remains a challenge.

**Scheme 1:**
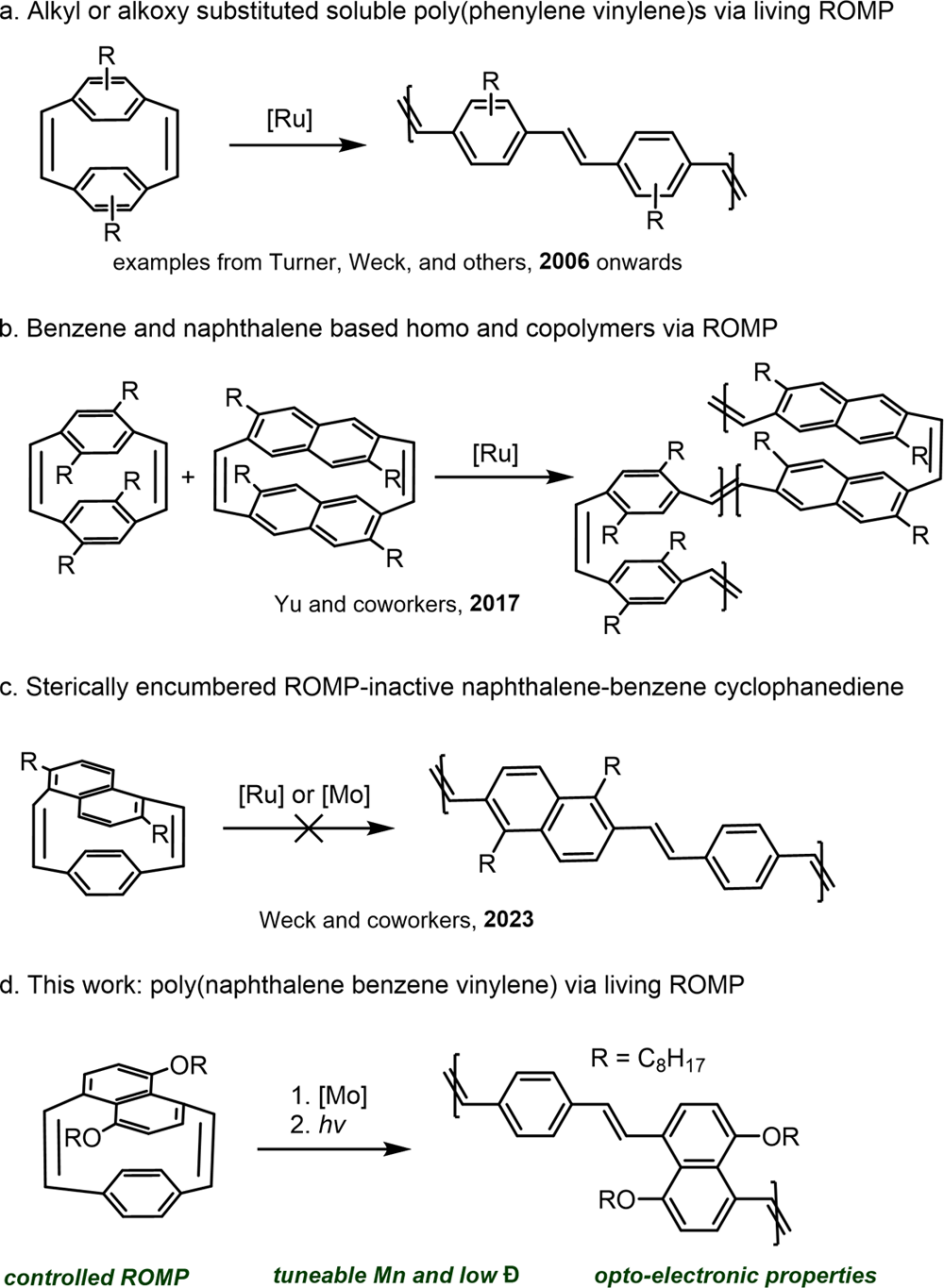
Recent Examples of the Ring-Opening Metathesis Polymerization (ROMP) of Cyclophanediene Analogues

**Figure 1:**
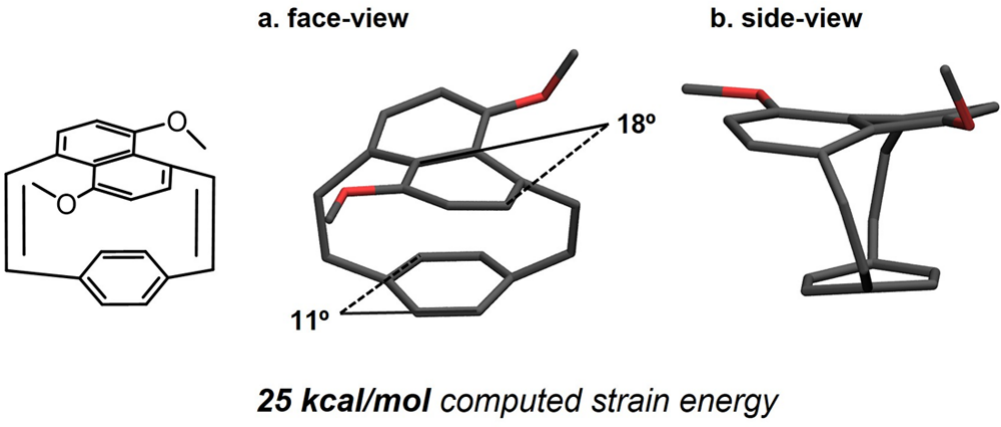
Optimized structure of monomer with truncated methoxy side chains obtained from DFT calculations showing (a) the front-view of the cyclophane diene with average dihedral twists along the four aromatic carbons next to the alkene bridges and (b) the side-view showing slipped geometry of the arenes. Hydrogens hidden for clarity.

Herein, we report on the synthesis of a ROMP-active mixed (1,5)-naphthalene-(1,4)-benzene cyclophane-2,5-diene (**6-M_NB_**) (Scheme 1d). Our cyclophanediene system features two alkene bridges connecting the rings at the (1,5) and the (1,4) positions on the naphthalene and the benzene sides, respectively. The alkoxy substitutions on the naphthalene ring are *para* to the diene substitutions, which we hypothesized would reduce steric crowding around the dienes allowing for catalyst coordination and polymerization. Structural information obtained from DFT calculations performed on **6-M_NB_** with truncated methoxy substituents indicates that there is at least one face free of steric bulk on either of the phane-dienes (Figure 1).^40^ Computed strain energy of 25 kcal/mol is on par with many ROMP-active norbornenes,^41^ indicating that **6-M**_**NB**_ would likely undergo ROMP to form poly­(naphthalene benzene vinylene)­s or PNVBs (Figure S.11). With this structural information on hand, we synthesized **6-M**_**NB**_ following the reported Boekelheide route for the synthesis of cyclophanedienes (Scheme 2) starting from alkoxy-substituted bis-bromomethyl naphthalene (**1**) and bis-methyl thiol benzene (**2**).^10,26,42,43^ Slow addition of **1** and **2** into KOH in ethanol over 70 h afforded the thioether bridged “di-thia phane” compound **3**. A benzyne induced Stevens rearrangement formed the bis-phenyl sulfide cyclophane product **4** as an inseparable mixture of diastereomers. Overnight oxidation with aqueous hydrogen peroxide followed by thermal elimination in refluxing xylenes afforded the desired monomer **6-M**_**NB**_ with ∼2% yield over four steps.

**Scheme 2:**
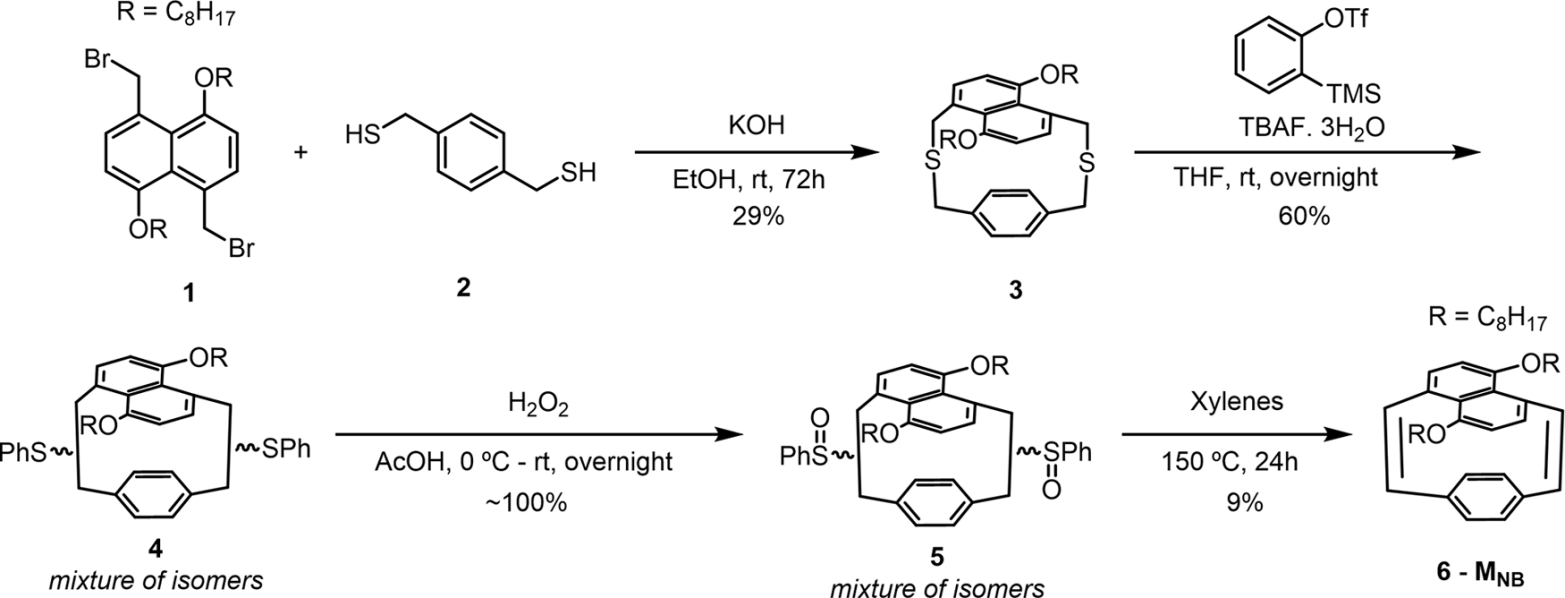
Synthesis of Cyclophanediene Monomer (**6-M_NB_**) Using the Boekelheide Route

Next, we investigated the ability of **6-M**_**NB**_ to undergo ROMP. Monomer **6-M**_**NB**_ undergoes ROMP with Grubbs II, Hoveyda-Grubbs II, and Grubbs III, but the polymerizations were sluggish in comparison to reported rates for the ROMP of benzene *para*-cyclophane dienes with ruthenium catalysts (Figure S.3).^10^ We attribute this slower polymerization behavior to the lower ring strain (25 kcal/mol) of **6-M**_**NB**_ as opposed to the >30 kcal/mol of strain commonly reported for paracyclophanedienes.^10^ Using the highly reactive molybdenum imido olefin metathesis catalyst developed by Schrock, we observed polymerizations of PNBV at faster rates (Scheme 3). Next, we investigated the controlled nature of molybdenum-catalyzed ROMP. We performed *in situ* ^1^H NMR spectroscopy experiments, monitoring the polymerization over the course of 24 h. We observed characteristic broadening of signals, along with near full conversion of the monomer when the polymerization achieved the target degree of polymerization (*n* = 20), as observed by ^1^H NMR end-group analysis (Figure S.2). Over time, the carbene signal shifts from 12.1 ppm to both 12.6 and 12.5 ppm with an eventual persistent carbene signal at 12.6 ppm (Figure 2c). The appearance of multiple downfield resonances in the carbene region is a typical indicator of the catalyst transformation from the inactive state to the active form and, in this case, attachment to the benzene or naphthalene sides with no obvious bias to either side. The persistence of the carbene resonance at the growing end of the polymer chain indicates the absence of a significant amount of chain-transfer or termination events, typically seen in living or highly controlled polymerizations.^19,44^ This result, combined with the observation of linearly increasing molecular weights with increasing monomer/initiator ratio (Figure 2a) and the corresponding decrease in GPC retention time with increasing chain length (Figure 2b), indicates that the polymerization proceeds in a highly controlled manner. While linearly increasing with [M/I], the 10-mer and 20-mer GPC traces show smaller *M*_n_ shoulders likely due to slower propagation that occurs due to decreased monomer concentration at high conversions, especially for higher [M/I] loadings. The errors are attributed to random operator error that accompanies measuring and dispensing miniscule amounts of catalyst inside a glovebox. MALDI-TOF analysis revealed major peaks that are 511 Da apart, the weight of a repeat unit, as expected with mass numbers suggesting a heterotelechelic end-group attachment of the *tert*-butylbenzene (from the catalyst) on one end and ethene (from the terminator) on the other end. While the major signals showcase end-group control, the presence of other signals suggests imperfections in end-group attachment likely from incomplete or undesired terminations from trace impurities in commercially sourced solvent or the terminator (Figure S.4).

**Scheme 3:**
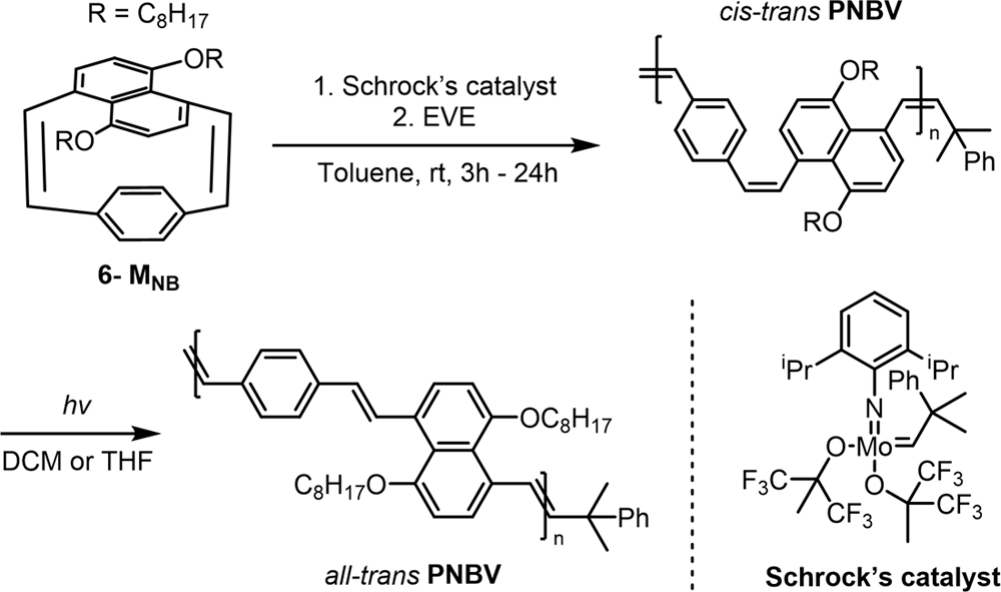
Polymerization of **6-M**_**NB**_ Using Schrock’s Molybdenum Catalyst and the *hv*-Induced Isomerization to Form *All-Trans* **PNBV**

**Figure 2:**
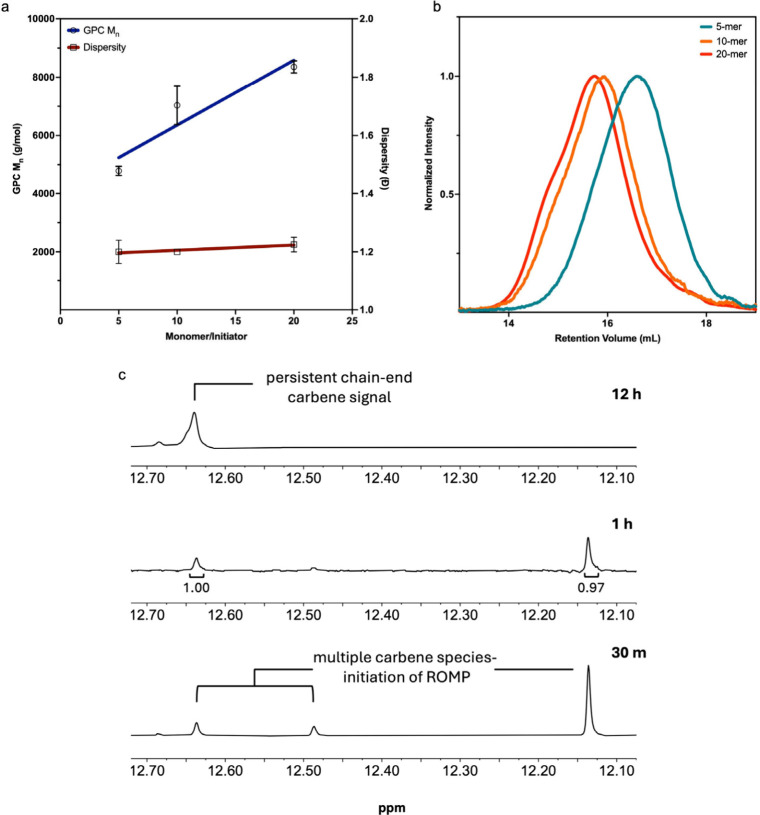
Living tests demonstrating controlled polymerization of PNBV. (a) Observed molecular weights (*M*_n_) and dispersity with changing monomer to initiator ratio. (b) GPC traces of corresponding [M]/[I] ratios. (c) In-situ ^1^H NMR experiment focusing on the carbene region over time.

Next, we characterized the optical properties of our PNBV system in solution and in the bulk. The absorbance and emission spectra of PNBV with different degrees of polymerizations were obtained in THF (Figure 3). The absorbance spectra of PNBV shows broad absorbance bands at 300–450 nm with an absorbance maximum at 390 nm. There is no significant difference between different chain lengths. On a representative 20-mer solution, we observed the characteristic red-shift in the absorbance maxima from the as polymerized “*cis–trans*” polymer to the UV-light isomerized “*all-trans*” polymer. The red shift in the absorbance maximum is the result of an increase in effective conjugation length and the subsequent decrease in the optical band gap.^30–32^ The emission spectrum of the polymers in THF excited at 393.5 nm was recorded and revealed two distinct, equally emissive peaks around 450 and 600 nm. We attribute these two peaks to the benzene (600 nm) and the naphthalene (450 nm) chromophores along the polymer backbone. This has been reported in block copolymer systems containing multiple aromatic chromophores in a single chain.^37^ The red-shifted emission maximum of the benzene chromophore is likely due to aggregation-induced emission aided by staggered pi interactions between polymer chains allowing for enhanced aggregation. This ability to access two distinct emission peaks may be useful for light emitting or fluorescence-labeling applications.^45,46^

**Figure 3:**
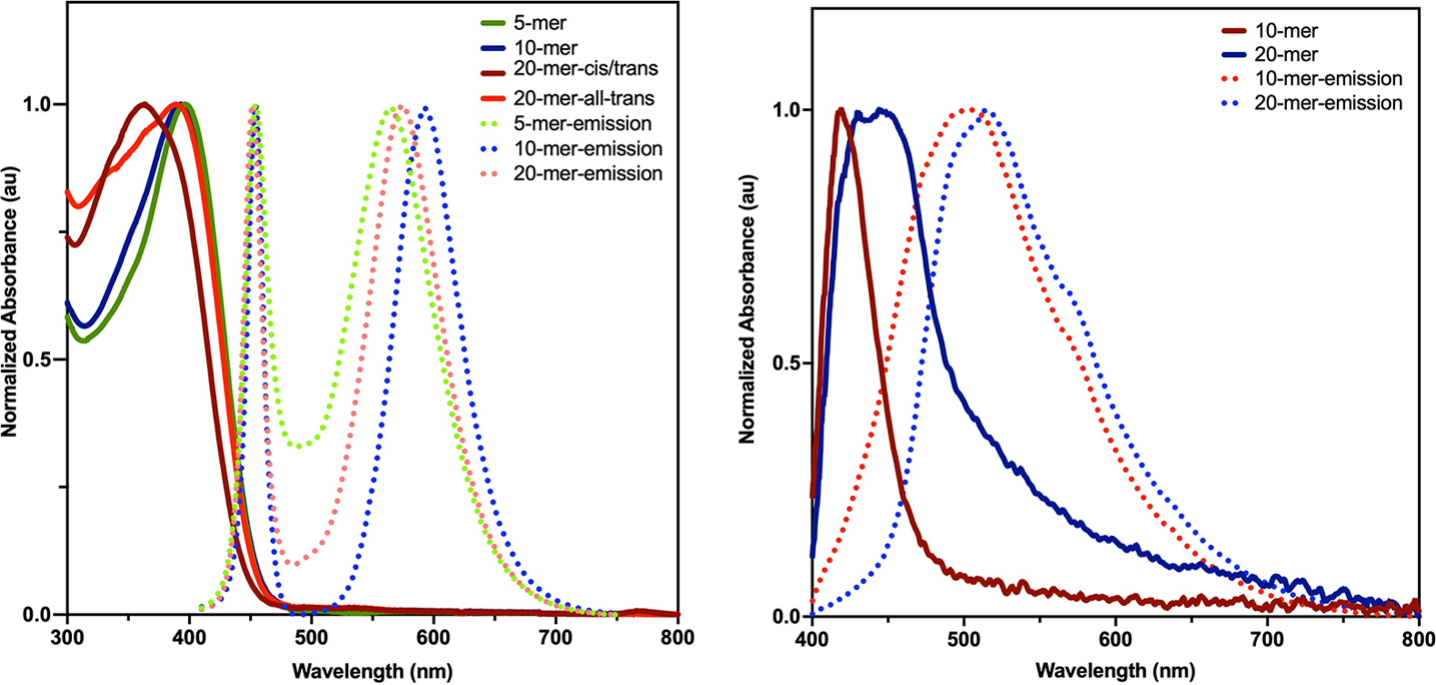
Absorbance and emission spectra at abs maxima for PNBV in THF. Cis/trans isomerization shown for a representative 20-mer (left). Absorbance and emission spectra of thin films of PNBV drop cast at 25 °C (right), λ_excitation_ = 365 nm.

Conjugated polymers are typically processed as semicrystalline or amorphous thin films for optoelectronics. Thermogravimetric analysis (TGA) of PNBV revealed the onset of decomposition at 140 °C and 25% mass loss by 365 °C (Figure S.5). Differential scanning calorimetry (DSC) showed a glass transition temperature at 56–61 °C, well below the onset of decomposition (Figure S.6). We characterized the absorbance and emissive signature of PNBV as thin films drop cast from dichloromethane or chloroform (Figure 3). The absorbance spectra obtained were red-shifted by ∼60 nm compared to the solution spectra in THF, with a maximum at ∼450 nm. The 20-mer film has a broad absorption tail extended to ∼800 nm while the 10-mer film exhibits narrower absorption with a slight tail extension to 700 nm. Upon excitation with 365 nm, the solid-state emission spectra showed a broad signature with the maximum around 525 nm. The two distinct emission peaks observed in solution were lost in bulk, presumably due to significant peak broadening from interchain interactions. The 20-mer has a slightly red-shifted absorbance and emission trace compared to the 10-mer. To test if the PNBV films had any thermal memory impacting bulk properties the absorbance and emission spectra of the films were taken while annealing at 60 °C over the course of 1 h (Figure S.10). The absorbance spectra for 10-mer and 20-mer films showed increased broadening; while the absorbance maxima remained unchanged. The emission spectra for 10-mer and 20-mer films showed no significant changes postannealing. The absence of any significant red shifting in the maximum wavelengths in the spectra suggests the lack of any significant thermal rearrangement that could affect device performance.

The PNBV films were then tested for their potential use as hole-transport materials (HTM)­s. Bottom-gate, bottom-contact transistors were fabricated on doped silicon with a 300 nm SiO_2_ layer with an active area of 7.5 × 10^–4^ cm^2^. The 10-mer PNBV polymers were drop cast from chloroform onto the transistor platform and allowed to dry at room temperature (Figure S.7). Doped transistors were prepared according to the SI, with FK 209 and Li-TfSI added to the solution before drop casting. For both devices, the source-drain voltage was swept from 0 to −20 V with a stepped gate bias from 0 V to −40 V in 10 V increments (Figure 4). Only a small response to the gate voltage was observed, with a hole mobility, μ, of 8 × 10^–9^ cm^2^/V·s extracted from the saturation regime. With the addition of commonly used p-type dopants (Li-TFSI and FK 209), μ increased to 1 × 10^–7^ cm^2^/V·s (Figure 4). This enhancement is attributed to increased hole carrier concentration typically observed upon doping organic semiconductors with Li-TFSI and FK 209.^47,48^ Overall, this increase in hole mobility indicates that the developed PNBV can be an attractive candidate for optoelectronics after further optimization.

**Figure 4:**
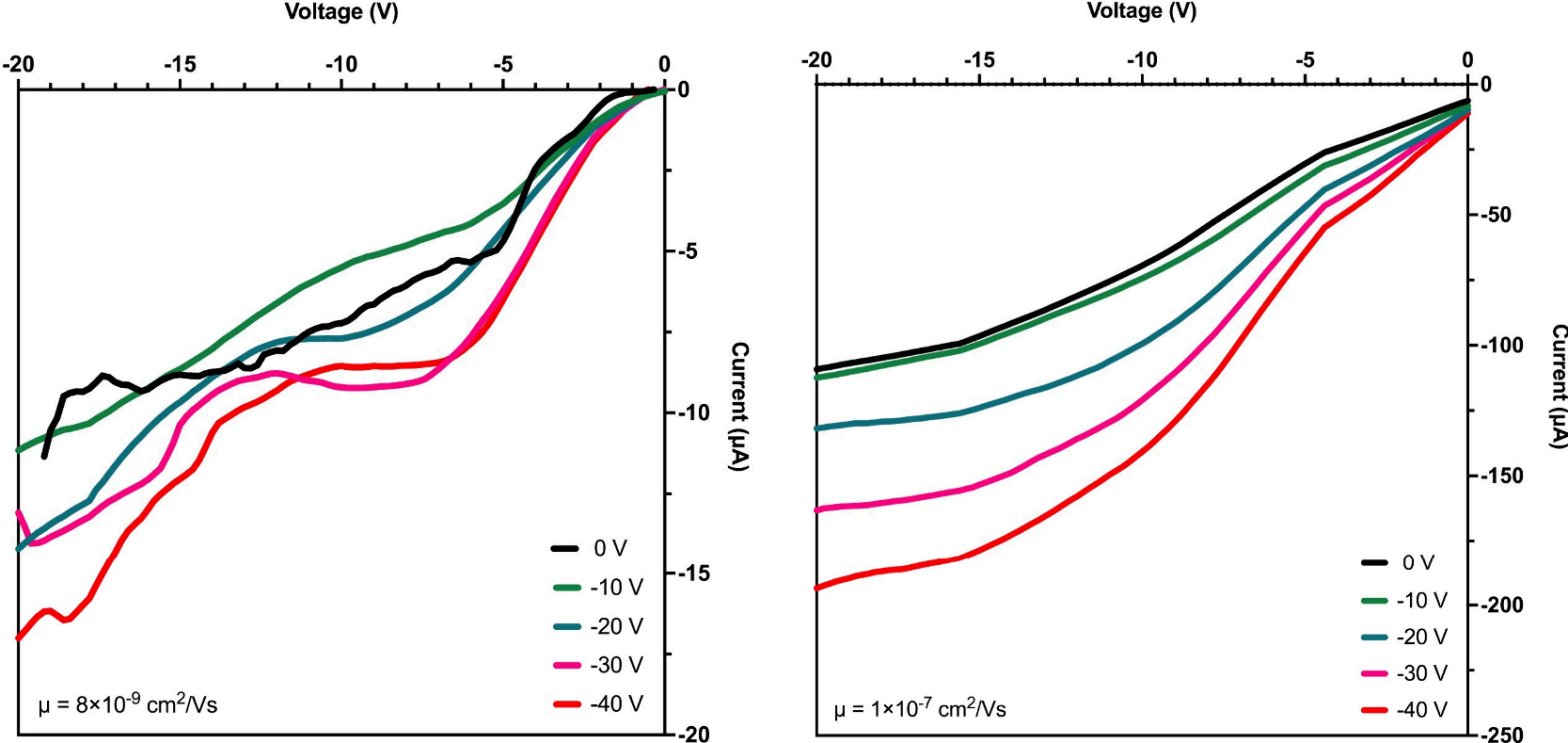
Charge transport measurements of undoped (left) and doped (right) PNBV transistors made from 10-mers. Measured current is across a source-drain voltage sweep of 0 V to −20 V at fixed gate voltages (*V*_g_).

In summary, we have synthesized a highly strained mixed cyclophanediene containing substituted naphthalene and benzene rings, demonstrated its controlled ROMP behavior with living characteristics, and characterized the physical and optoelectronic properties of the resulting PNBVs. The monomer, **6-M**_**NB**_, has a ring strain of 25 kcal/mol similar to the ROMP-inactive monomer reported earlier by us.^38^ We demonstrate that tuning the placement of the substituents on the naphthalene ring alleviates steric hindrance around the alkene allowing catalysts to coordinate and initiate ROMP. This was aided by structural computational analysis of potential monomers using DFT which, in part, guided monomer design. We anticipate this not only highlights the role of substitution placement in ROMP of naphthalene-benzene cyclophanedienes but can guide future monomer design in similar systems. Additionally, the emissive and conducting nature of the PNBVs films indicates that these polymers could be developed for optoelectronics. Overall, our strategy provides access to polymers with larger, fused rings, and differing aromatic repeat units in a living manner with complete control over basic polymer properties. We anticipate this methodology will enable access to electronically diverse π-conjugated polymers with control, i.e., chain growth synthesis of third generation semiconducting polymers.^4–6^

## Supplementary Material



## Notes

Full experimental details, NMR spectra, physical characterizations, and computational details (PDF)
