# Supplementary material for: Strain, Chain, Repeat: Synthesis and Optoelectronic Properties of Poly(Naphthalene Benzene Vinylene)s
Source: ACS Macro Lett. 2026 Jun 3;15(6):864–70. doi: 10.1021/acsmacrolett.6c00194 (PMC13276905; doi:10.1021/acsmacrolett.6c00194)
Supplement: Supplementary file 1 [file mz6c00194_si_001.pdf]

Supporting Information

Strain, Chain, Repeat: Synthesis and Optoelectronic Properties of Poly(Naphthalene Benzene Vinylene)s

*Bibek Dhungel, Mia Klopfenstein, Anahita Keer, Matthew D. Hannigan, Susanna Monore, Jordan M. Garber, Wandy Rodriguez, Stephanie Lee, and Marcus Weck**

Molecular Design Institute and Department of Chemistry, New York University, NY, USA

*To whom correspondence should be addressed: marcus.weck@nyu.edu

Material and Methods 2

Polymerizations 3

2.1. Typical polymerization using Schrock's [Mo] catalyst 3

2.2 In situ ¹HNMR polymerization using Schrock's Catalyst 3

2.3 Results of in-situ ¹HNMR using Schrock's Mo catalyst 4

2.3 Polymerizations using Grubbs catalysts 6

2.4. Results of in-situ ¹HNMR polymerizations using Grubbs catalysts 6

Physical Characterizations 7

3.1. Results of MALDI TOF Analysis 7

3.2. Results of Thermogravimetric Analysis (TGA) 8

3.3. Results of Differential Scanning Calorimetry (DSC) measurements 9

3.4. Electronic characterization of thin films 9

3.5. Optical Micrographs of drop-cast polymer films 10

3.6. Optical data on thin films with thermal annealing 11

Small Molecule Synthesis 12

4.1 NMR Spectra 15

Computational Details 20

5.1. Results of strain energy calculations 20

5.3. XYZ coordinates of optimized structures 21

References 26

Material and Methods

All chemicals were purchased from Ambeed, Thermo Fisher, Oakwood Chemicals, TCI Chemicals, Sigma Aldrich, Strem or other commercial vendors and used as received unless otherwise indicated. All reactions were carried out under ambient conditions unless otherwise noted. Flash column chromatography was performed using silica gel 60 Å (230-400 mesh) from Sorbent Technologies.

NMR spectroscopy was performed on Bruker Advance 400 MHz, 500 MHz 600 MHz or 800 MHz spectrometers. Chemical shifts are reported in ppm and referenced to residual solvent peaks.[1] Splitting patterns are reported as singlets (s), doublets (d), doublet of doublets (dd), triplets (t), quartets (q) or multiplets (m). Spectra were collected under ambient conditions in $CDCl_3$ unless otherwise noted.

High resolution mass spectra of filtered samples in methanol or ethanol were performed with an Agilent 6224 LC-TOF Mass Spectrometer on ESI+ mode. GCMS was performed with a Shimadzu TQ8040 GCMS in Electron Impact (EI) mode. MALDI- Time of Flight readings were taken in Bruker autoflex maX spectrometer using Dithranol or DCTB matrices. MALDI samples were prepared by mixing 1mg/mL solution of polymers in DCM with a 5 mg/mL solution of matrix in DCM in a 1:1 ratio.

Gel-Permeation Chromatography (GPC) traces were obtained from a Shimadzu pump coupled to a Shimadzu UV and RI detectors with tetrahydrofuran (THF) mobile phase. The injection volume was 50- 100 µL and the flow rate was 1mL/min on a Shimadzu column set (100, 1000, 100,000 Å, linear mixed bed). GPC calibrations were performed with poly(styrene) standards (EasiCal, Agilent Technologies, Santa Clara, CA). All characterizations were performed at 40 ºC.

Absorption spectra were obtained in dilute solutions of THF using an Agilent Cary 3500 UV-vis at 25 ºC. Fluorescence spectra were collected in dilute solutions of THF on a Horiba PTI QuantaMaster 400 Fluorometer at 25 ºC. Thermogravimetric Analysis were performed on a Waters Discovery Thermogravimetric Analyzer 5500 by TA instruments. Measurements were taken from 25 ºC to 800 ºC using 100 µL platinum pans. Differential Scanning Calorimetry was performed on a PerkinElmer DSC 8000.

Graphical analysis and statistics were performed using Prism 11 by GraphPad. Reported error-bars are standard error of the mean on duplicate measurements. Computations were performed at the DFT level of theory on Gaussian 16[2] using the Greene High Performance Computing Cluster at New York University. Optimized structures were visualized on Visual Molecular Dynamics (VMD 1.9.3)[3] using ACS recommended settings. Reported bond angles, distances, dihedral angles, or other properties were calculated using VMD or Avogadro[4] on optimized structures.

Polymerizations

2.1. Typical polymerization using Schrock's [Mo] catalyst

Polymerizations using Schrock's [Mo] catalyst (2,6-Diisopropylphenylimidoneophylidene molybdenum (VI) bis(hexafluoro-t-butoxide)) were performed in a nitrogen filled glovebox. In a typical procedure, 5-6 mg of the monomer **(6-M_{NB})** was weighed out in a scintillation vial equipped with a stir bar and an air-tight Teflon-septa cap and dissolved in 200-400 µL of anhydrous toluene. Appropriate amounts of the Schrock's catalyst (based on target M/I ratios) were weighed out, dissolved in anhydrous Toluene and added to the monomer solution. Reactions at 5 mg scale were performed with a total of ~500 µL anhydrous toluene. The reaction was covered in foil and left to stir inside the glovebox at room temperature for 6-24 hours (approximately one hour for every repeat unit) in the dark. Upon completion, degassed Ethyl vinyl ether (EVE) (20 eq. to the catalyst) was added using a syringe through the septa, and the solution was left to quench for two hours. The mixture was then brought out of the glove box and the solvent evaporated leaving behind a green or brownish film. To this residue, cold methanol (5 mL) was added, and the solution was vigorously sonicated and shaken using a vortex machine to completely suspend the particles. The resulting suspension was passed through a short plug of celite and washed with more cold methanol to remove excess terminator. Finally, the plug was washed with THF to dissolve the residual polymer, which was collected separately. The obtained polymers were analyzed by GPC, MALDI-TOF, UV-Vis, and Fluorescence spectrometer without further purification.

Samples used in physical characterization as thin films were additionally purified by dialysis over three days using suspensions in acetone utilizing 1 kDa exclusion membranes. Samples used to collect *hv* induced cis-trans isomerization data were kept in the dark strictly throughout the polymerization and the workup.

2.2 *In situ* ^{1}HNMR polymerization using Schrock's Catalyst

In situ ^{1}HNMR experiments were performed using a J-Young NMR tube equipped with an airtight cap. In a nitrogen atmosphere glovebox, 5-6 mg of monomer **(6-M_{NB})** were dissolved in 300 µL of anhydrous deuterated toluene (Toluene-d8) and added to a J-Young tube. Appropriate amount of Schrock's olefin metathesis catalyst was weighed out in the glovebox, dissolved in 200 µL of Toleune-d8 and added to the monomer solution. The tube was sealed, removed from the glovebox and immediately covered in foil and kept cold over an ice bath to slow the polymerization. The tube was then inserted into a Bruker Advance 600 MHz NMR spectrometer and ^{1}HNMR spectra were collected over time (typically every 20 minutes over 24 hours) at 25 ºC. After completion, the reaction mixture transferred to a scintillation vial in the glovebox and quenched with EVE. The reaction was worked up as described above.

2.3 Results of *in-situ* ^{1}HNMR using Schrock's Mo catalyst

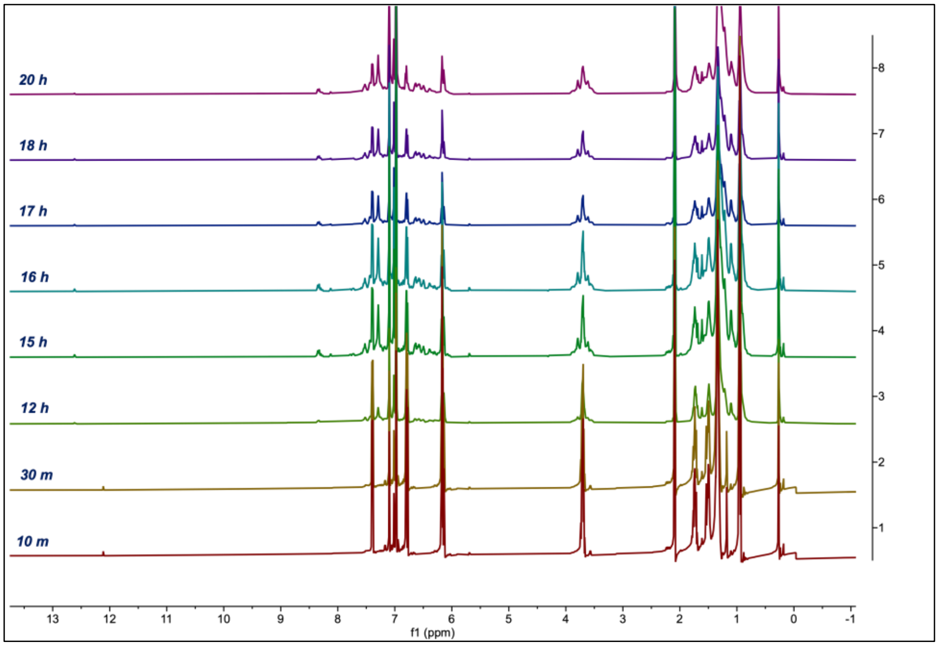

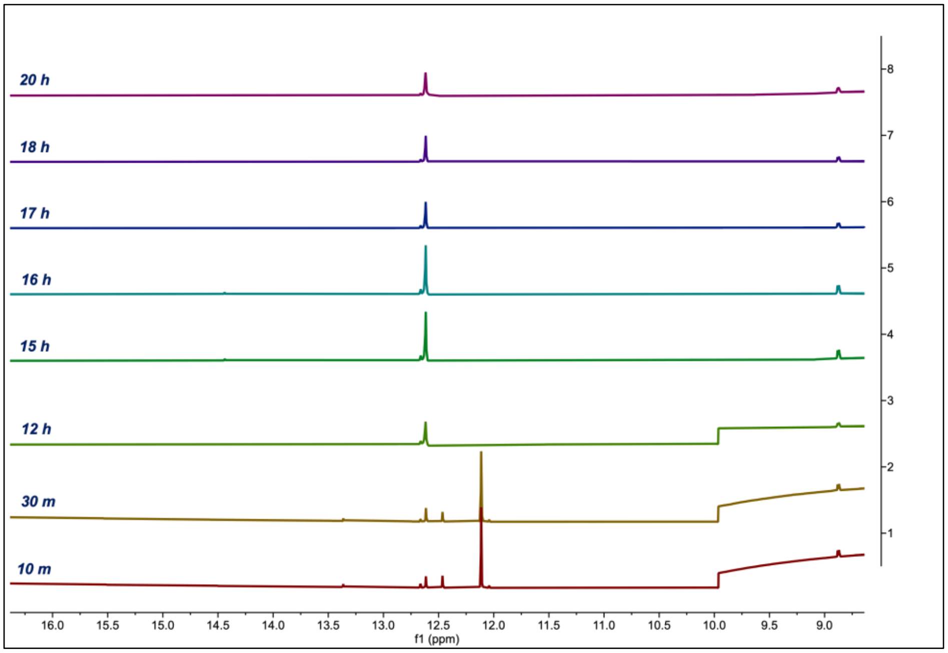

Figure S.1. *In-situ* ^{1}HNMR spectrum of a representative 20-mer showing broadening polymer signals over time (top). Expanded carbene region for the same polymerization showing catalyst activation and persistence over time (bottom).

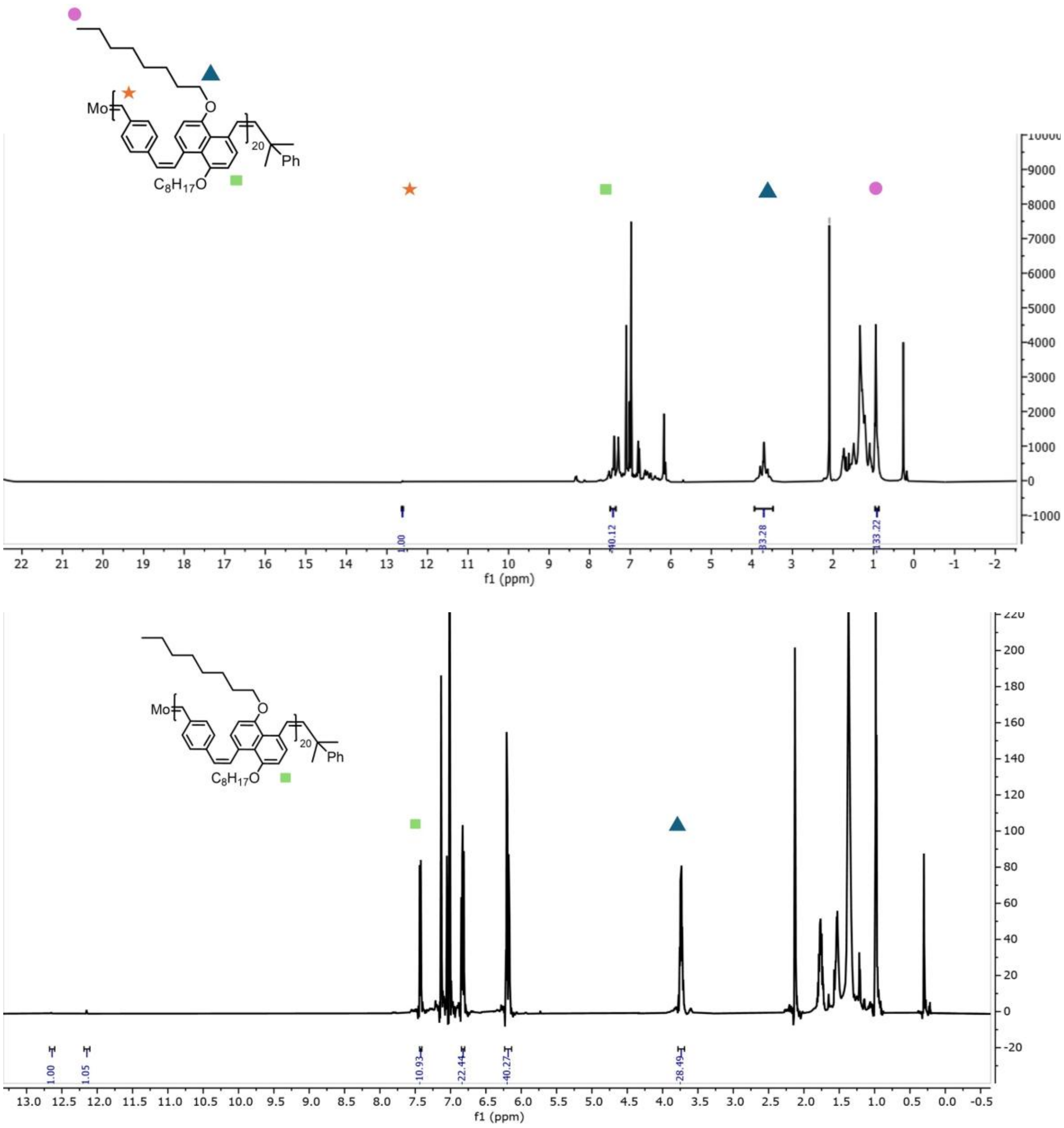

Figure S.2. End-group analysis of PNBV 20-mer in Toluene-d^8 20 hours after initiation (**top**) and 30 minutes after initiation (**bottom**). Carbene resonance is normalized to 1 (Mo=CH proton) and integrated against the discernable and distinct proton signals (40H, 83H, 133H) indicating a n = 20 (**top**). Two carbene resonances seen during initiation are each 1:1 and normalized to 1H. Residual monomer signals integrate to 11H, 22H, 40H, and 28H; ½ of M/I = 20 as expected due to 1:1 carbene signals (**bottom**).

2.3 Polymerizations using Grubbs catalysts

Polymerizations using the Grubbs catalysts were performed at the same concentration and following the same general procedure as polymerizations with the Schrock's catalyst. *In-situ* ^{1}HNMR Polymerization using the Hoveyda Grubbs II and Grubbs 2nd generation catalysts were performed in THF-d^{8} at 45 ºC.

2.4. Results of *in-situ* ^{1}HNMR polymerizations using Grubbs catalysts

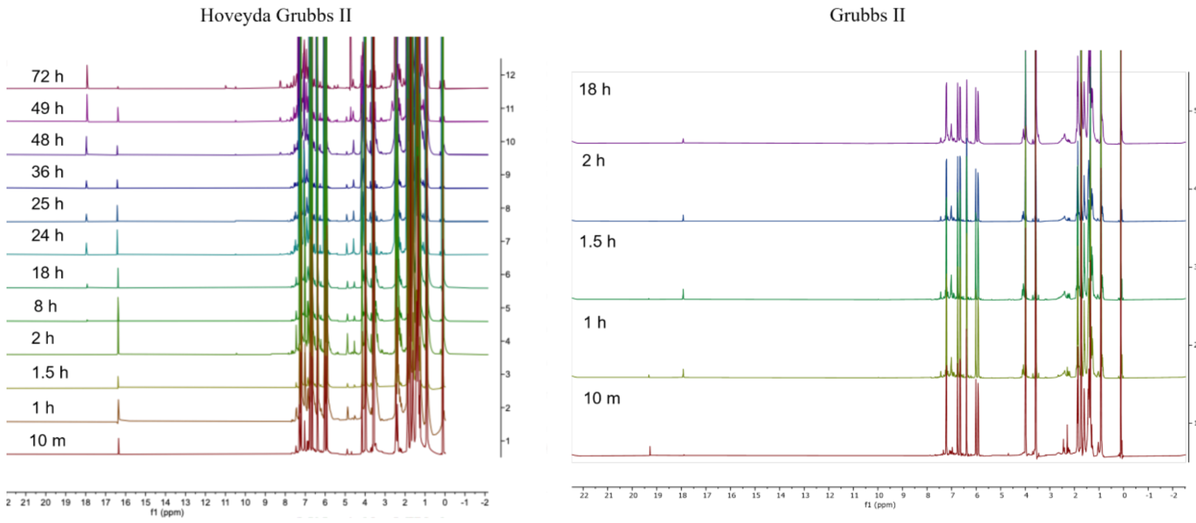

Figure S.3. *In-situ* ^{1}HNMR spectrum of polymerizations using Hoveyda Grubbs II catalyst showing incomplete imitation over the course of 72 hours (**left**). *In-situ* ^{1}HNMR Polymerization using Grubbs II catalyst showing fast initiation but minimal monomer conversion over the course of 18 hours (**right**).

Physical Characterizations

3.1. Results of MALDI TOF Analysis

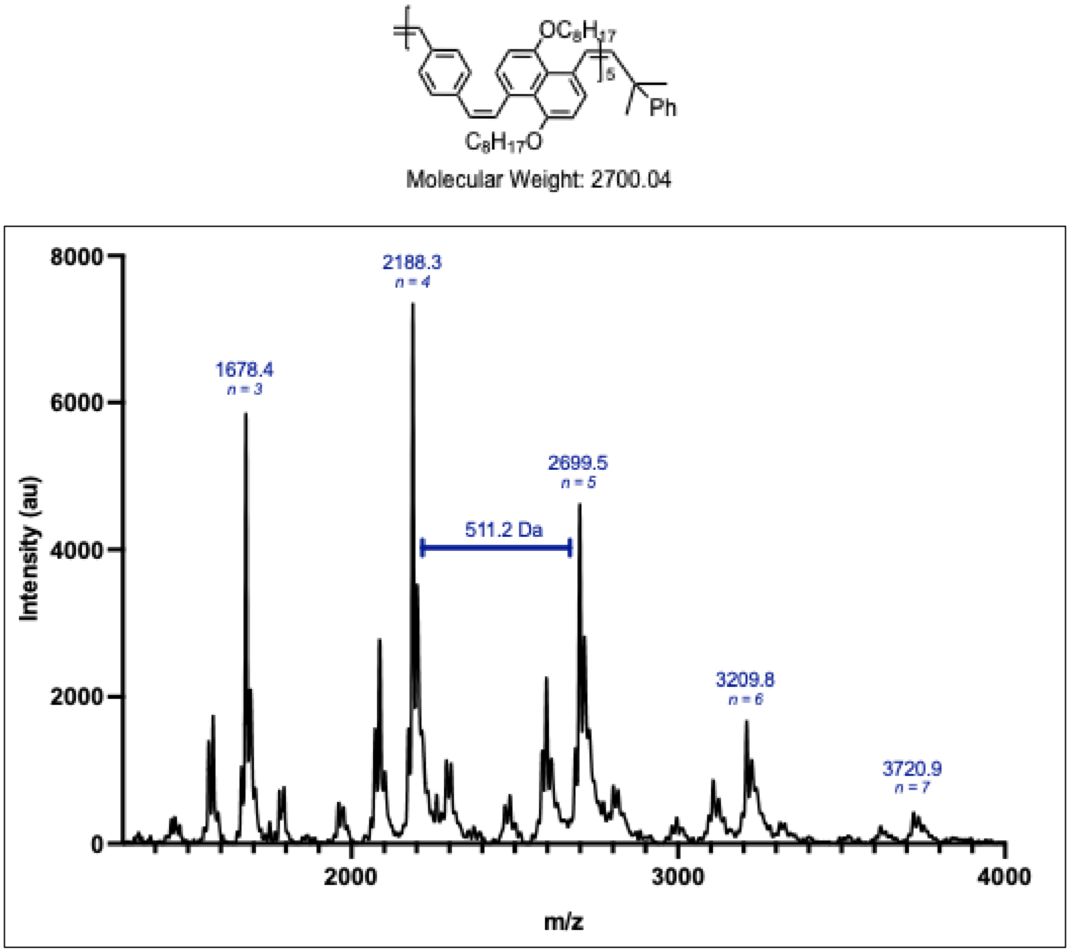

Figure S.4. MALDI TOF trace of a representative 5-mer. Major peaks are ~511 Da apart which is close to the mass of every repeat unit (510.76 Da) and correspond to n = 3 – 7. Major m/z ratios correspond to end-group attachment as depicted.

3.2. Results of Thermogravimetric Analysis (TGA)

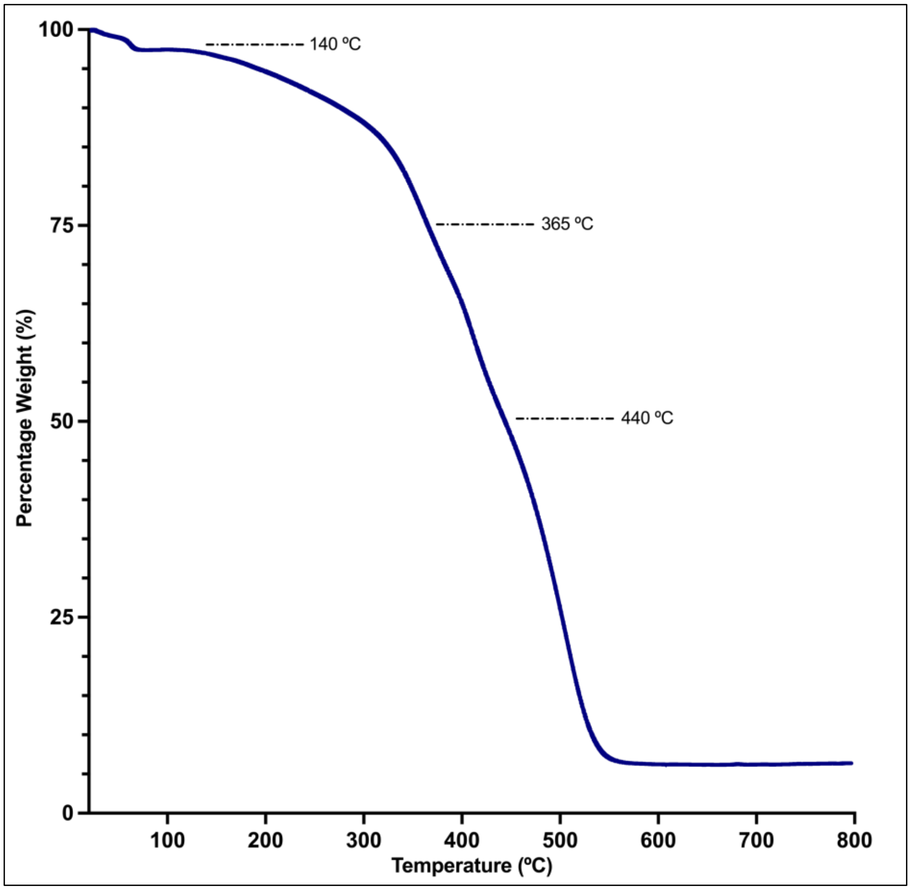

Figure S.5. TGA trace of a representative 20-mer. Onset of decomposition is around 140 ºC, 25% weight loss is seen at 365 ºC, and 50% weight loss is seen by 440 ºC.

3.3. Results of Differential Scanning Calorimetry (DSC) measurements

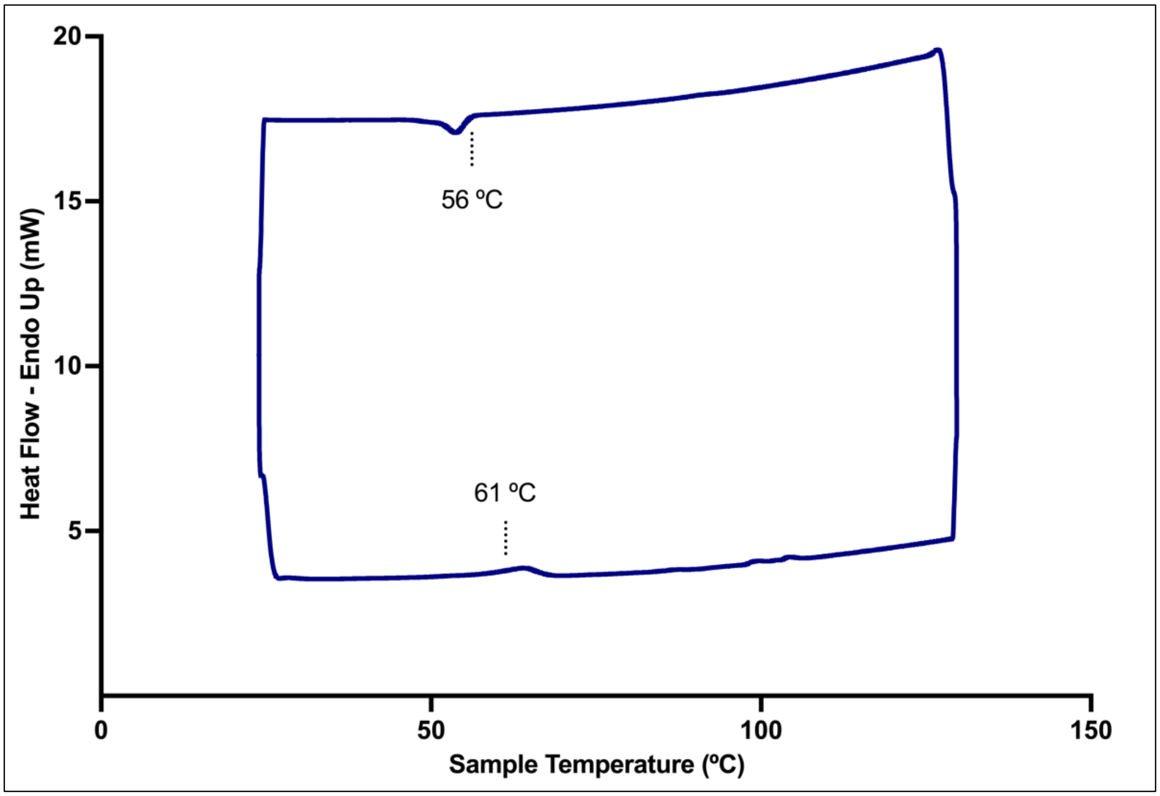

Figure S.6. DSC trace of a representative 5-mer showing the glass transition temperature Tg between 56-61 ºC. Depicted measurement is the second run following an initial run using the same parameters.

3.4. Electronic characterization of thin films

Transistor Fabrication: Silicon wafer with 300 nm oxide layer was cut to size and sonicated with acetone, methanol and DI water for ten minutes each and dried with compressed air. 10 nm of Cr and 100 nm of Au were thermally evaporated at 0.1 and 0.5 nm s-1 onto the Si wafer through a shadow mask to define coplanar electrodes (active area = 0.00075 cm2). Undoped device preparation: 20 µL of 10-mer polymers from 10mg/mL in CF were drop cast onto prepared substrates. Doped device preparation: 10-mer polymers from 10mg/mL in CF was doped with bis(trifluoromethylsulfonyl)imide lithium salt (Li-TFSI, 20 mL, 517 mg/mL in acetonitrile) and tris(2-1H-pyrazol-1-yl)-4-tertbutylpyridine)cobalt(III) tri[bis(trifluoromethane)sulfonimde] (FK 209, 8 ml, 376mg/mL in acetonitrile). After mixing, 20 µL of solution was drop cast onto prepared substrates and allowed to dry at RT to form a solid thin film. Doped polymer transistors were tested a day after drop casting.

Electronic characterization: Transistor I-V curves were collected at room temperature with a digital source meter (Keithley model 2636B). The applied voltage was swept from 0 to -20V with a gate bias of 0 V, -10 V, -20 V, -30 V and -40 V.

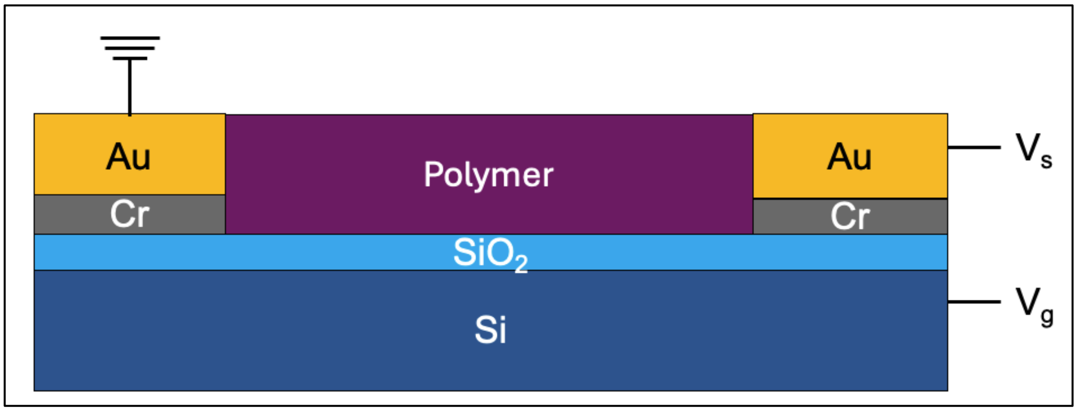

Figure S.7. Schematic representation of devices fabricated for use in charge transport measurements.

3.5. Optical Micrographs of drop-cast polymer films

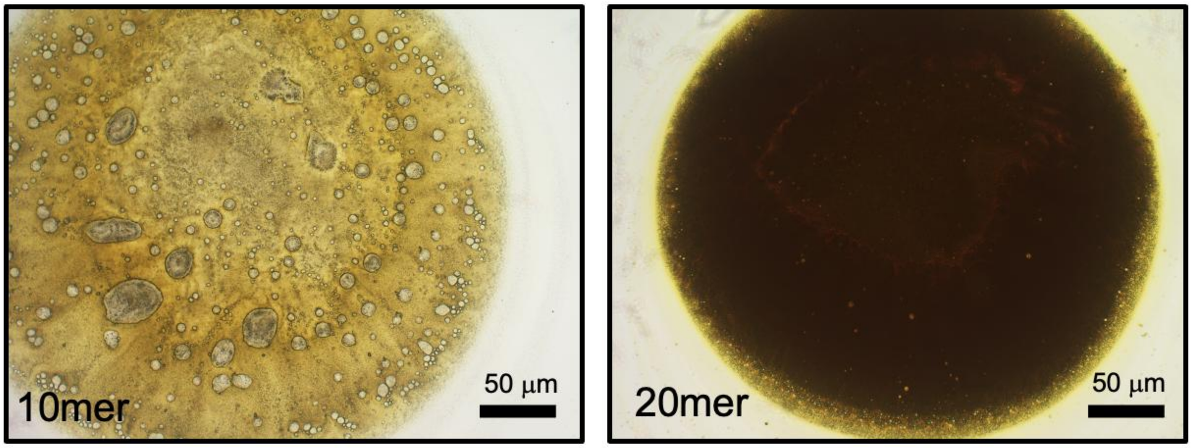

Figure S.8. Optical micrographs of drop cast 10-mer (left) and 20-mer (right) films. Films were fabrication by drop casting 20 μL of 10 mg/mL solution from CF.

3.6. Optical data on thin films with thermal annealing

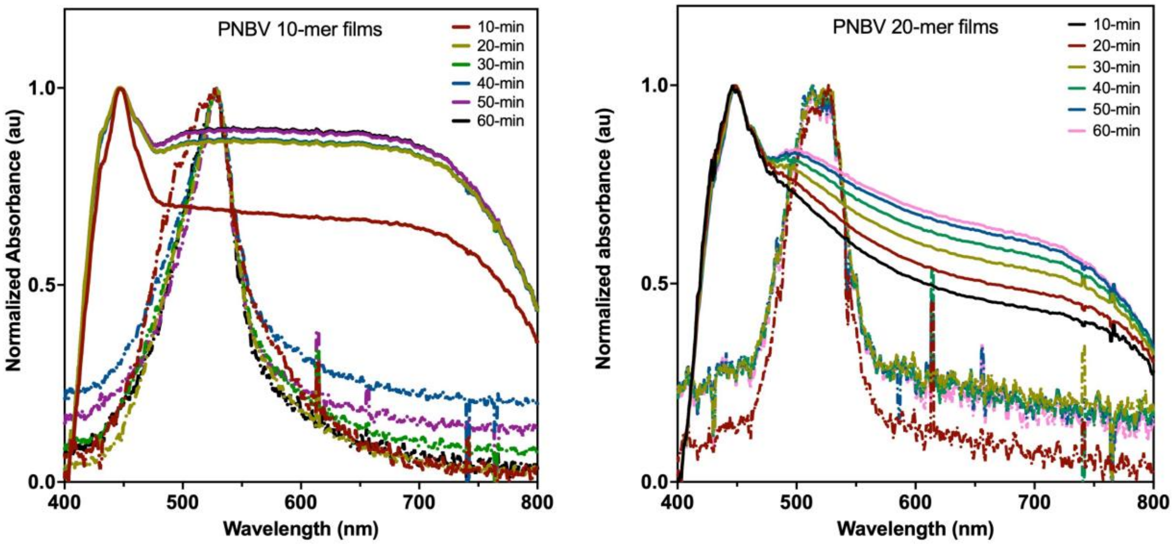

Figure S.10. Emission (**bold**) and absorbance (**dash**) spectra of PNBV thin films taken during thermal annealing at 60 ºC over the course of 1 hour. Both 10 and 20-mer films show no significant changes or red-shifting during annealing that would indicate improved performance induced by thermal rearrangement.

Small Molecule Synthesis

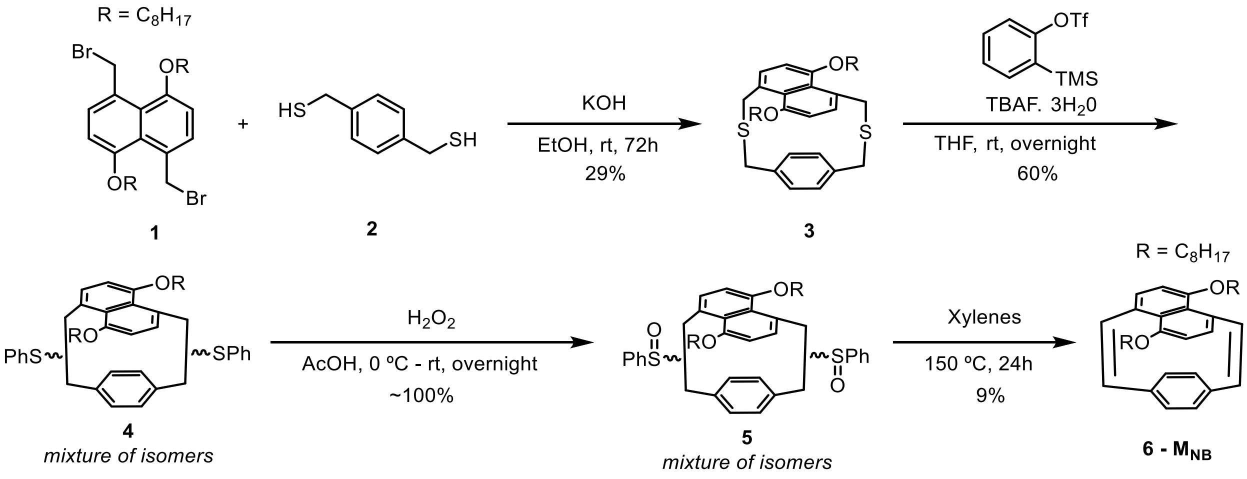

1,5-Bis(bromomethyl)-4,8-bis(octyloxy)naphthalene (1)

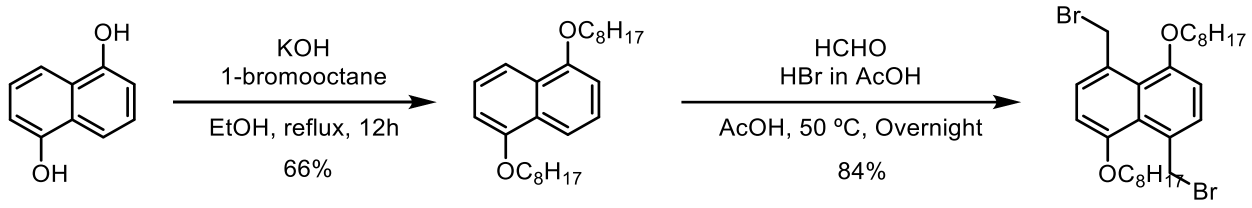

1

Compound **1** was synthesized in two steps following modified literature procedures.[5]

Napthalene-1,5-diol (10.0g, 62 mmol), KOH (10.5g, 187 mmol), and ethanol (200 proof, 200 mL) were added to a round bottom flask equipped with a stir bar. Octyl bromide (24 mL, 137 mmol) was slowly added, and the mixture was refluxed for 12 hours. The resulting mixture was cooled to room temperature, and the solids collected after washing with water and ethanol. The collected solids (16.0g, 66%) were used in the next step without further purification.

1,5-Bis(octyloxy)naphthalene (16.0g, 42 mmol), paraformaldehyde (2.87g, 96 mmol), and glacial acetic acid (100 mL) were added to a round bottom flask equipped with a stir bar. The resulting suspension was cooled over an ice bath, and HBr (33% in Acetic Acid, 60 mL, 0.36 mol) was added dropwise. The mixture was then heated to 50 ºC for six hours. The mixture was then cooled to room temperature, and the sediment was filtered, followed by successive washes with copious amounts of saturated sodium bicarbonate and water. Further drying under vacuum resulted in a greyish-brown amorphous solid **1** (20.0g, 84%).

^{1}HNMR shifts match reported spectra.[5] ^{1}H NMR (400 MHz, $CDCl_3$) δ 7.38 (d, J = 8.2 Hz, 2H), 6.85 (d, J = 8.2 Hz, 2H), 5.32 (s, 4H), 4.14 (t, J = 6.7 Hz, 4H), 2.12 – 1.99 (m, 4H), 1.61 – 1.49 (m, 8H), 1.47 – 1.21 (m, 12H), 0.94 – 0.83 (m, 6H)

1,4-Phenylenedimethanethiol (2)

Compound **2** was synthesized following modified procedures from the literature.[6] 1,4-Bis(bromomethyl)benzene (5.00 g, 19 mmol), thiourea (3.20 g, 42 mmol) and ethanol (60mL, 200 proof) were added to a round bottom flask equipped with a stir bar. The mixture was degassed with nitrogen for 30 minutes- then heated to reflux for 6 hours under nitrogen. The reaction mixture was then cooled to room temperature, and the solvent was evaporated to *in vacuo*. The resulting white salts were dissolved in aqueous NaOH (5M, 30mL), degassed for 30 minutes with nitrogen, and refluxed overnight under nitrogen. The solution was cooled to 0 ºC and slowly acidified to pH ~2 by addition of 12M HCl. The solids were collected by filtration, then washed with water and dried. The dried solids were suspended in hexanes washed with copious amounts of hexanes to filter out insoluble solids. Evaporation *in vacuo* resulted in the desired product as white crystalline solids in quantitative yields.

^{1}HNMR shifts match reported spectra.[6] ^{1}H NMR (400 MHz, $CDCl_3$) δ 7.28 (s, 4H), 3.73 (d, J = 7.5 Hz, 4H), 1.75 (t, J = 7.5 Hz, 2H).

Dithia[3.3]-1,5-napthalenoparacyclophane (3)

KOH (3.93g, 56 mmol) was dissolved in ethanol (200 proof, 700 mL) in a three-neck neck flask equipped with a stir bar and an addition funnel with an adjustable bore metering plug. Compounds **1** (4.00g, 7 mmol) and **2** (1.19g, 7mmol) were dissolved in toluene (500 mL) and added to the addition funnel. Both solutions were degassed for 30 minutes and then left under a nitrogen atmosphere. The toluene solution was slowly added to the ethanol solution over a course of at least two days at a rate of about 1 drop every 5-10 seconds. After addition, the reaction was left to stir for an additional 24 hours before concentrating the mixture *in vacuo* resulting in a brown slurry. This slurry was taken up in DCM, filtered through a short plug of silica, and washed with copious amounts of DCM. The organic layers were evaporated *in vacuo.* The residue was then adhered to *celite*, and the mixture was purified via flash chromatography on silica gel using a gradient eluent system of 10% – 30% DCM in hexanes, giving compound **3** as a green oil that solidifies into waxy green solids upon standing. (1.20g, 29%) HRMS ESI Calculated for $C_{36}H_{50}O_2S_2$: 578.3252, found [M+Na]+: 601.3126

^{1}H NMR (500 MHz, $CDCl_3$) δ 7.09 (d, J = 7.9 Hz, 2H), 6.54 (dd, J = 7.8, 2.0 Hz, 2H), 6.50 (d, J = 8.0 Hz, 2H), 6.10 (dd, J = 7.8, 2.0 Hz, 2H), 4.93 (d, J = 14.0 Hz, 2H), 4.05 (dt, J = 8.9, 6.4 Hz, 2H), 4.01 – 3.93 (m, 4H), 3.62 (d, J = 14.9 Hz, 2H), 3.39 (d, J = 14.9 Hz, 2H), 1.92 (m, J = 13.3, 8.5, 4.7 Hz, 4H), 1.66 – 1.11 (m, 20H), 0.90 (t, J = 6.8 Hz, 6H).

^{13}C NMR (126 MHz, $CDCl_3$) δ 156.5, 136.7, 129.3, 127.7, 127.0, 126.8, 126.6, 105.2, 77.4, 77.2, 76.9, 68.6, 39.8, 36.0, 32.0, 29.6, 29.5, 29.4, 26.6, 22.8, 14.3.

Bis(sulfide)-1,5-napthalenoparacyclophane (4)

Using Schlenk technique, compound **3** (2.00 g, 3.5 mmol) was added to a flame-dried three-neck flask equipped with a stir bar and an addition funnel under a flow of nitrogen. Anhydrous THF (80 mL) was added to dissolve the solids followed by 2-(Trimethylsilyl) phenyltrifluoromethanesulfonate (2.10 mL, 8.6 mmol). Tetrabutylammonium fluoride hydrate (4.36g, 13.8 mmol) was dried under vacuum for four hours, dissolved in anhydrous THF (30 mL), and added to the addition funnel. The TBAF solution was added

dropwise to the stirring solution on the flask over four hours and left to stir overnight. The reaction mixture was concentrated, adhered to celite and purified by flash chromatography over silica gel using a gradient eluent system of 10% - 40% DCM in hexanes to afford compound **4** as a green oil. (1.53g, 60%) Mixture of isomers used without further purification. HRMS ESI Calculated for $C_{48}H_{58}O_2S_2$ 730.3878, found [M+Na]+: 753.3781

Bis(sulfoxide)-1,5-napthalenoparacyclophane (5)

Compound **4** (1.00g, 1.4 mmol) was dissolved in toluene (35 mL) and cooled to 0 ºC. Glacial acetic acid (11 mL) was added slowly followed by hydrogen peroxide (655 µL, 32%). The reaction was slowly warmed up to room temperature and left to stir overnight. The resulting mixture was diluted with DCM and brine, and extracted three times with DCM, followed by a wash with water, sodium bicarbonate, and brine. The organic layers were combined, dried over magnesium sulfate, filtered, and concentrated *in vacuo* to obtain the product, compound **5**, as a green oil in quantitative yields. Mixture of isomers used without further purification. HRMS ESI Calculated for $C_{48}H_{58}O_4S_2$: 762.3777, found [M+Na]+: 785.3725

1,5-Napthalena-(1,4)-benzenecyclophanediene (M-NpBn)

Using Schlenk technique, compound **5** (1.04g, 1.4 mmol) was dissolved in anhydrous xylenes and degassed for 30 minutes with nitrogen. The reaction flask was then covered in aluminum foil and refluxed (~140 ºC) under a flow of nitrogen for 24 hours. The mixture was then cooled to room temperature and concentrated in vacuo. The resulting oil was taken up in *celite* or silica and purified by flash chromatography on silica gel using a gradient eluent system of 10%-50% DCM in hexanes to afford monomer **M-NpBn** as a green oil that solidifies on standing (60 mg, 9%) HRMS ESI Calculated for $C_{36}H_{46}O_2$: 510.3498, found [M+Na]+: 533.3386.

^{1}H NMR (500 MHz, $CDCl_3$) δ 7.25 (d, J = 10.9 Hz, 2H), 6.82 (dd, J = 7.6, 1.2 Hz, 2H), 6.70 (d, J = 9.9 Hz, 2H), 6.36 (d, J = 7.6 Hz, 2H), 6.06 (dd, J = 7.7, 1.8 Hz, 2H), 5.98 (dd, J = 7.8, 1.7 Hz, 2H), 3.97 (dtd, J = 15.6, 9.0, 6.4 Hz, 4H), 1.86 (m, 4H), 1.61 – 1.51 (m, 4H), 1.49 – 1.16 (m, 16H), 0.95 – 0.88 (m, 6H).

^{13}C NMR (126 MHz, $CDCl_3$) δ 155.7, 136.8, 136.4, 131.5, 129.0, 127.5, 127.4, 126.1, 103.2, 77.4, 77.2, 76.9, 68.5, 32.0, 29.6, 29.5, 29.5, 26.6, 22.9, 14.3.

^{1}H NMR (500 MHz, Tol) δ 7.39 (d, J = 9.8 Hz, 2H), 6.87 – 6.68 (m, 4H), 6.25 – 6.03 (m, 6H), 3.77 – 3.59 (m, 4H), 1.83 – 1.61 (m, 4H), 1.49 - 1.34 (m, 20H), 0.95 (t, J = 6.7 Hz, 6H).

4.1 NMR Spectra

^{1}HNMR spectra of **3**

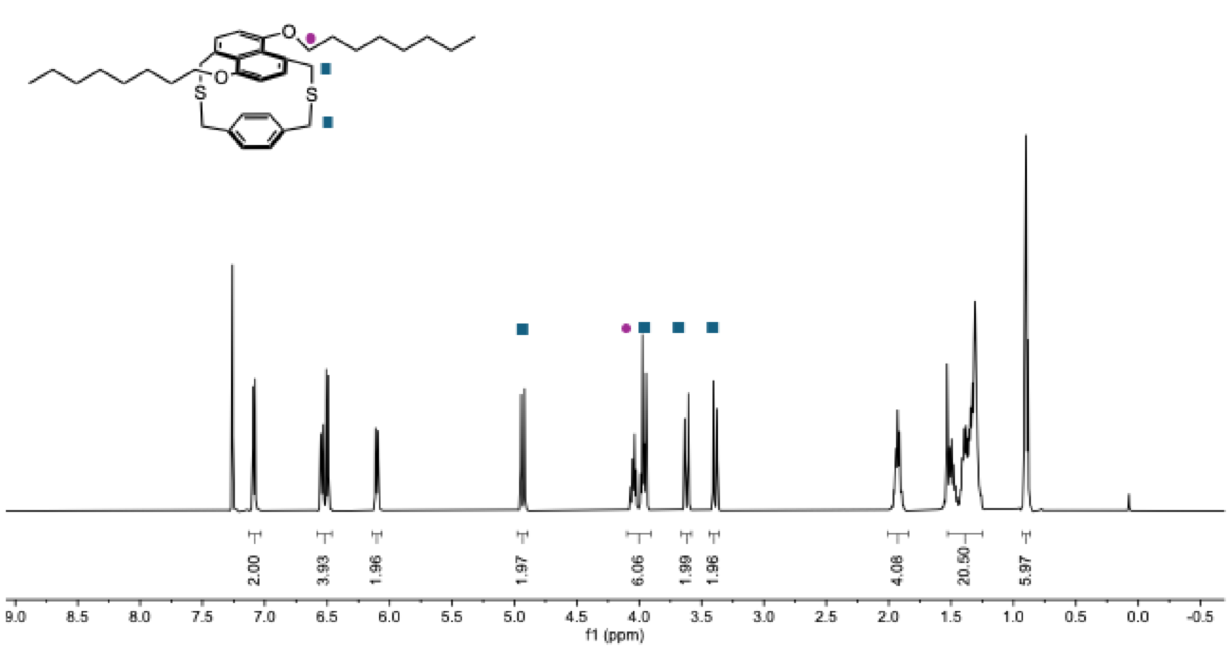

^{13}CNMR spectra of **3**

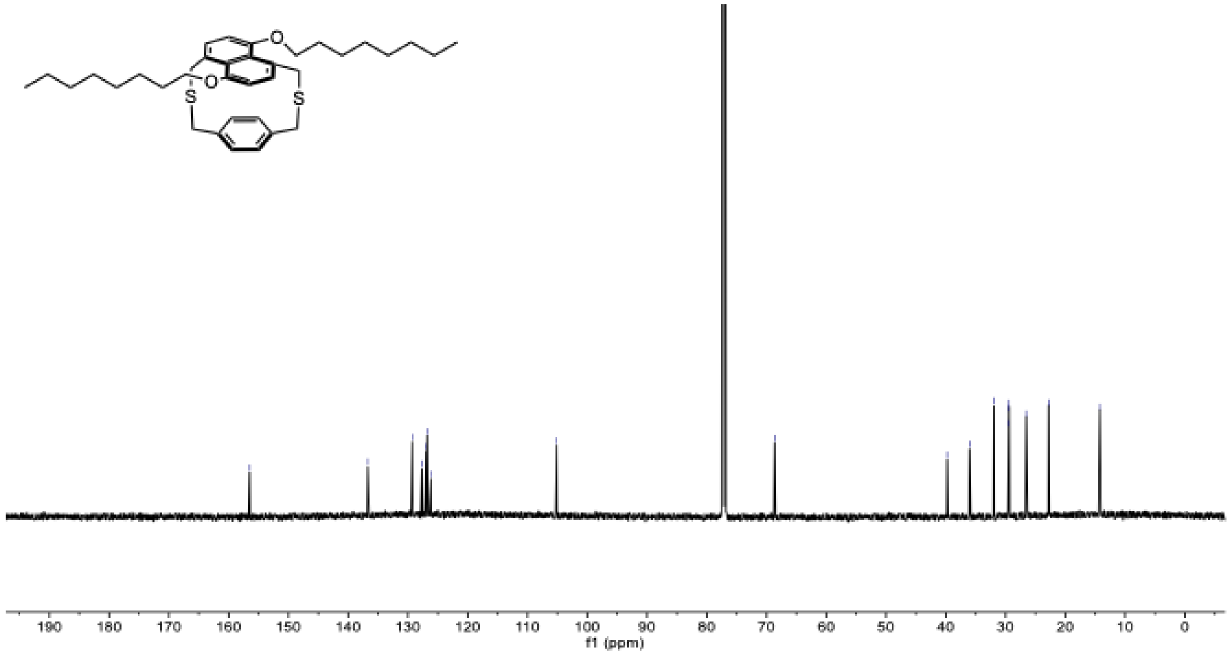

HSQC of **3**

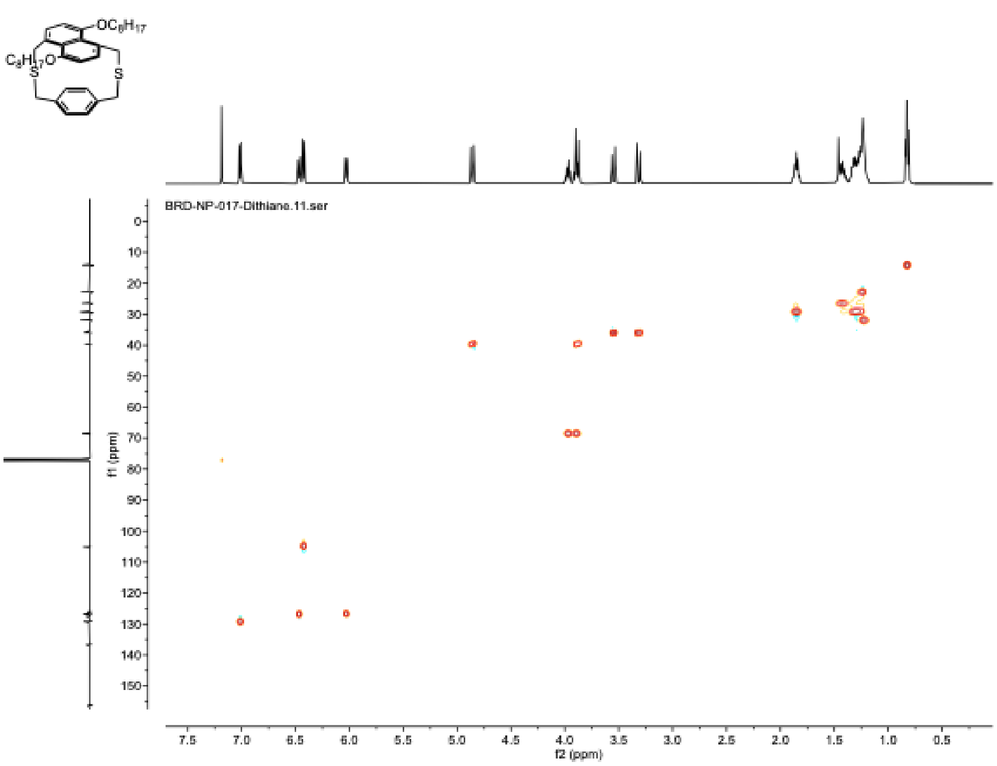

^{1}HNMR of **4**

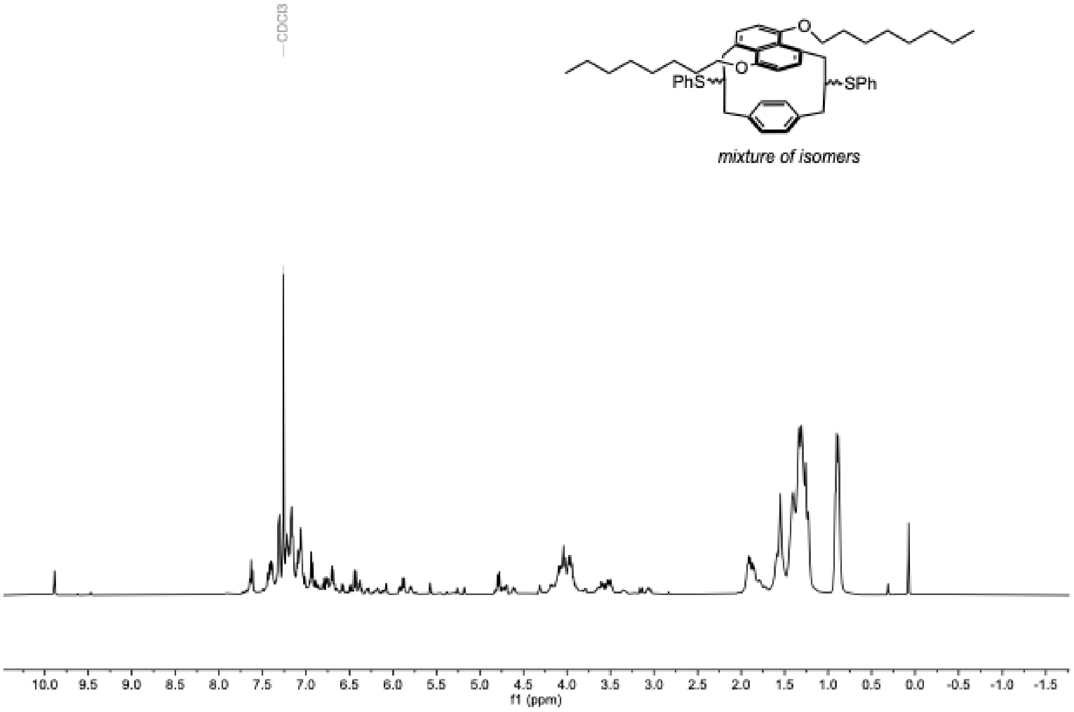

^{1}HNMR of **5**

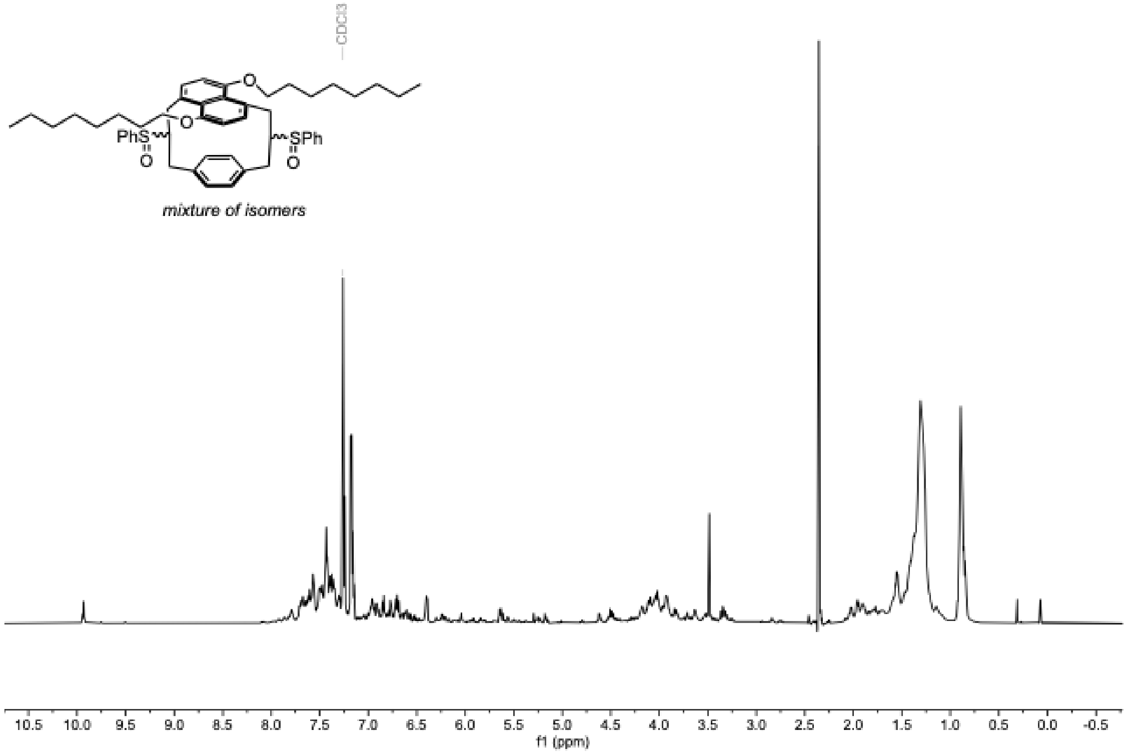

^{1}HNMR of **6-M$_{NB}$** in Toluene-d8

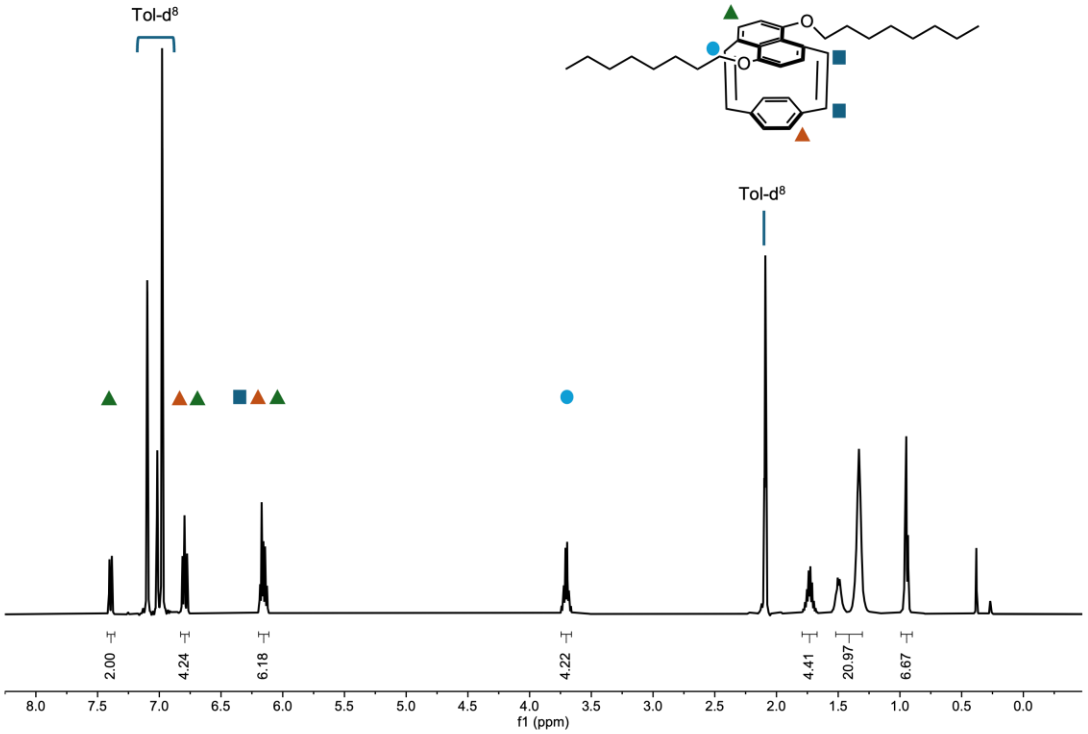

^{1}HNMR of **6-M$_{NB}$** in $CDCl_3$

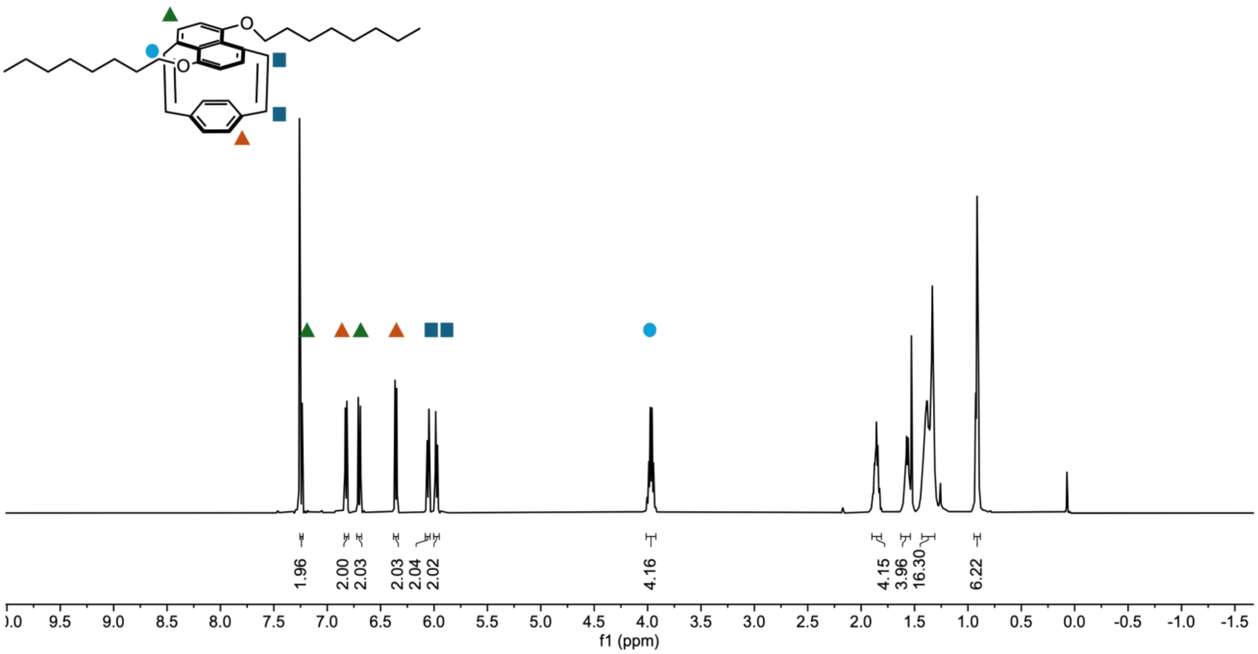

^{13}CNMR of **6-M$_{NB}$** in $CDCl_3$

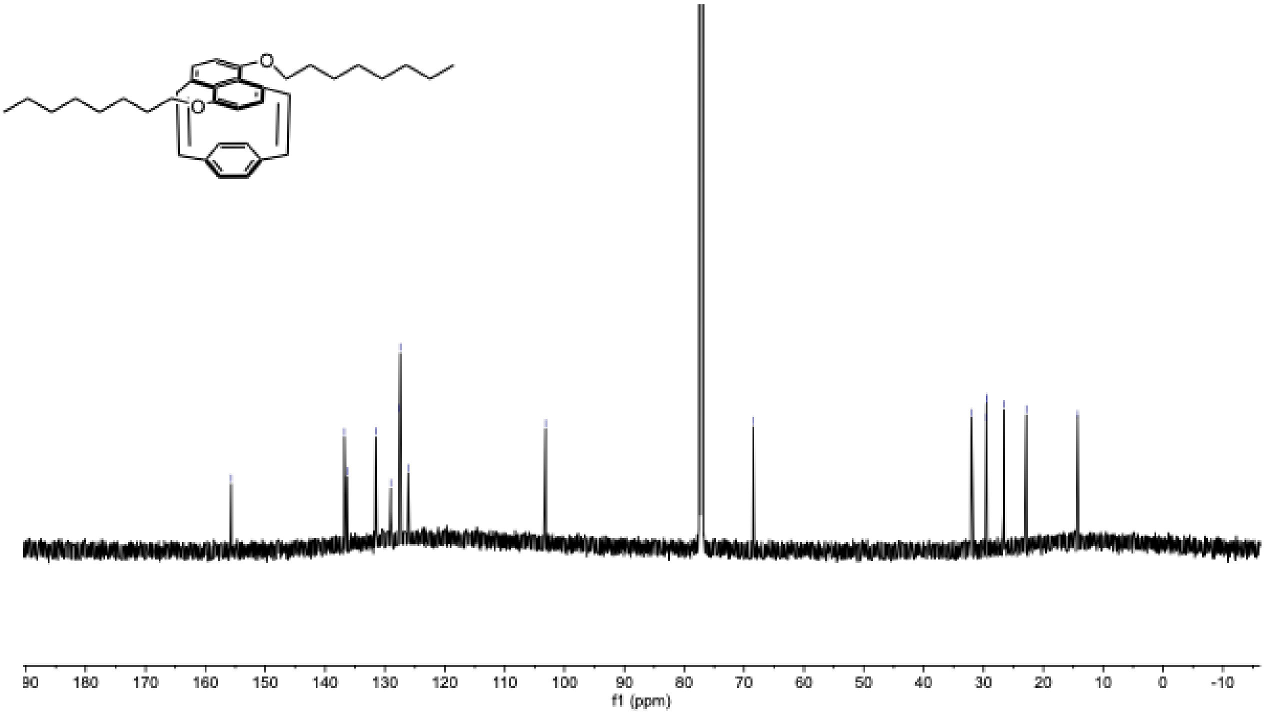

HSQC of **6-M$_{NB}$** in $CDCl_3$

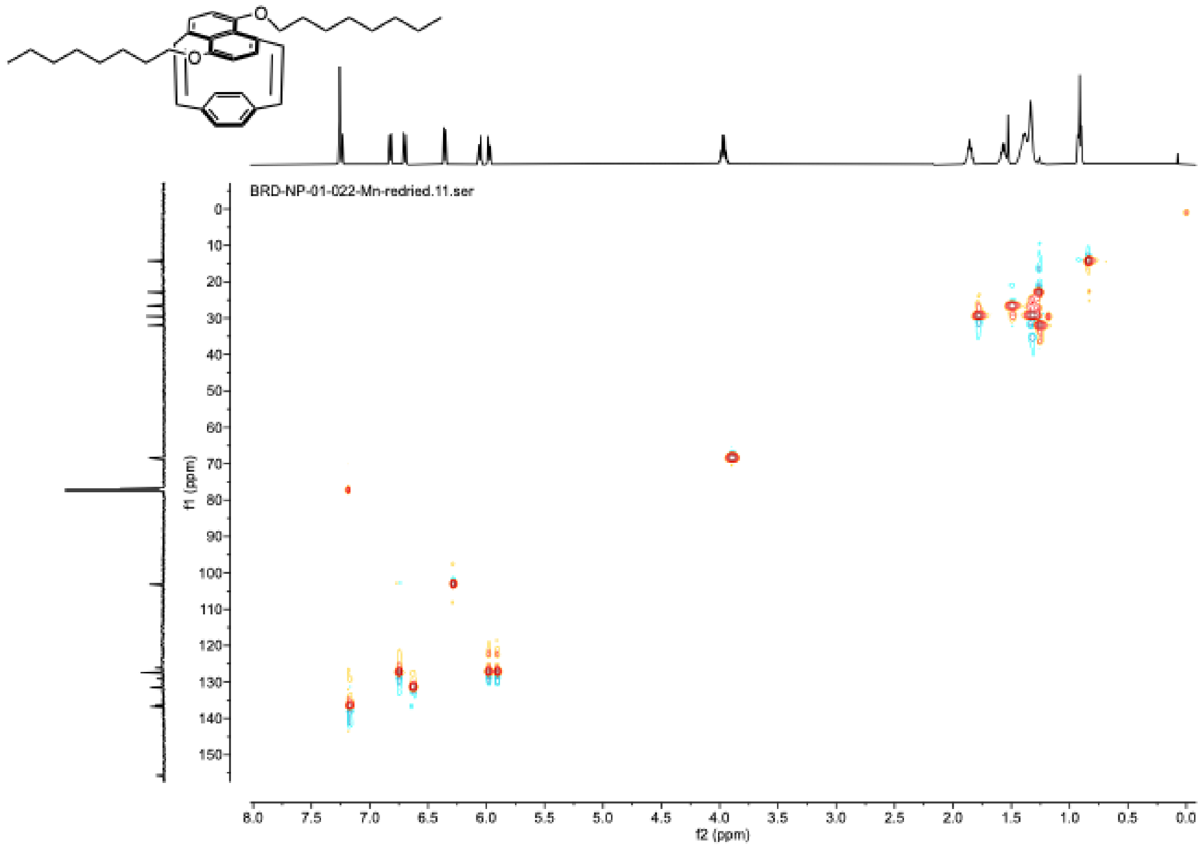

Computational Details

Calculations were performed at the DFT level of theory in Gaussian 16. In a typical workflow, initial molecular geometries were first obtained from Avogadro and minimized using the Uniform Force Field (UFF).[7] These XYZ coordinates were further optimized in Gaussian based on the M11 functional and LANL2DZ basis set a singlet spin sate.[8-10] The minimized energies of these structures were calculated as single point energies using the M11 functional and 6-311G* basis set.[8-10] These parameters were chosen based on earlier benchmarking reported by the Weck group on strain energy computations of cyclophane dienes using DFT.[11]

Strain energies for cyclophane dienes were on the heat of etheneolysis ($\Delta G_{ethenolysis}$) which is expected to closely match ring strain (ΔG_{strain}).[12-14] In a typical ROMP mechanism, strained cyclophane dienes such as **M1** go from the closed ring form to the open **M1-eth** form, allowing us to approximate:

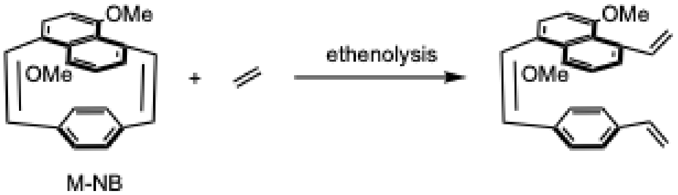

$$\Delta G_{strain} = - \Delta G_{ethenolysis}$$

$$\Delta G_{strain} = - \Delta G_{ethenolysis} = \Delta E_{MNB\text{-}Eth} - (\Delta E_{ethene} + \Delta E_{MNB})$$

Further optimizations and corrections were not performed, and the computed values are reported as close approximations.

5.1. Results of strain energy calculations

The following single point energies were computed for the open/closed version of M-NB

**ethene**	**-78.5411530317 Ha**
**M-NB-Closed**	**-999.153633335 Ha**
**M-NB-Open**	**-1077.73546838 Ha**

25 kcal/mol strain energy was calculated based on these values for **6-M_{NB}** truncated methoxy side chains.

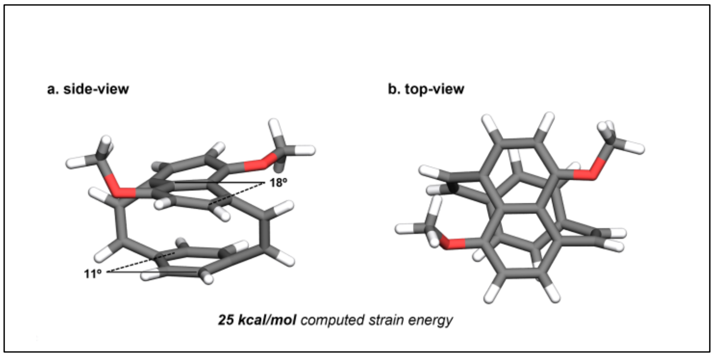

Figure S.11. Optimized structure of **6-M$_{NB}$** used in strain energy calculations also shown in the main text. The dihedral twists depicted correspond to the four aromatic carbons directly next to the alkene bridges.

5.3. XYZ coordinates of optimized structures

XYZ files are provided below in the input format for commonly used molecular editors that can read .xyz files such as Molden or Avogadro.

The format for the XYZ coordinates provided below is as follows:

First line = atom count (ex. 6 for ethene)
Second line = name of the molecule corresponding to the coordinates
Third line onwards = the element, x-coordinate, y-coordinate and z-coordinate

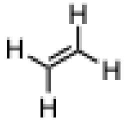

6

ethene

C	-2.184022	2.586350	-0.996287
C	-0.859078	2.772710	-1.113093
H	-2.710010	2.784029	-0.060597
H	-2.788235	2.229703	-1.832341
H	-0.254864	3.129357	-0.277039
H	-0.333090	2.575031	-2.048783

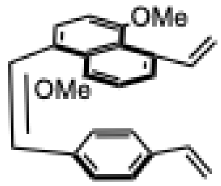

48

M-NP-Open

C	-7.260971	1.126244	3.050805
O	-6.439906	1.208198	1.867222
C	-5.065972	1.186186	2.003048
C	-4.449511	1.132616	3.238887
C	-3.039071	1.207614	3.321316
C	-2.235731	1.419687	2.211962
C	-0.769652	1.400017	2.515008
C	0.263767	0.759511	1.928548
C	0.291654	-0.071340	0.704647
C	1.511326	-0.202901	0.010370
C	1.593643	-0.944632	-1.173264
C	0.459481	-1.590908	-1.701677
C	-0.751571	-1.496582	-0.984673
C	-0.833636	-0.757698	0.198763
C	-2.886724	1.586198	0.928954
C	-4.291233	1.315614	0.790553
C	-4.888813	1.252270	-0.524660
C	-6.207858	0.645146	-0.872479
C	0.588666	-2.354283	-2.967004
C	-4.129387	1.658984	-1.610209
C	-2.789509	2.088493	-1.476265

C	-2.178166	2.041834	-0.237535
O	-0.887333	2.481344	-0.039069
C	-0.023671	2.635399	-1.186568
H	-8.294602	1.155135	2.695920
H	-7.072965	1.980501	3.720632
H	-7.081529	0.185441	3.595472
H	-5.032048	1.038665	4.153170
H	-2.564339	1.093763	4.297322
H	2.401579	0.293484	0.403266
H	2.547759	-1.027295	-1.698795
H	-1.639847	-2.015795	-1.346832
H	-1.773297	-0.728387	0.748583
H	-4.574101	1.619605	-2.606052
H	-2.245124	2.435872	-2.352261
H	0.981178	2.796099	-0.784257
H	-0.323217	3.503666	-1.794864
H	-0.032467	1.719261	-1.800977
H	1.243513	0.892609	2.399502
H	-0.527667	1.940923	3.435850
C	-6.712199	-0.492539	-0.367825
H	-6.733572	1.135175	-1.698198
H	-6.215339	-1.027012	0.443943
H	-7.645826	-0.912177	-0.744805
C	-0.410758	-2.935733	-3.655887
H	1.608209	-2.431094	-3.358828
H	-1.451467	-2.887622	-3.330026
H	-0.214766	-3.478927	-4.580424

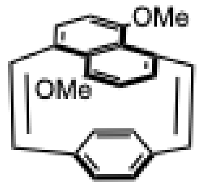

42

M-NB-Closed

C	-5.500569	1.050648	3.230838
O	-4.691057	1.513648	2.128486
C	-3.314913	1.502833	2.275816
C	-2.672500	1.160915	3.450783
C	-1.248168	1.061962	3.458931
C	-0.489673	1.456646	2.377800
C	0.892691	0.881637	2.184176
C	1.037323	-0.172545	1.346195
C	-0.169222	-0.660081	0.591299
C	-0.375835	-0.246595	-0.736942
C	-1.672869	-0.214054	-1.272205
C	-2.781616	-0.589542	-0.490673
C	-2.540480	-1.219551	0.744045
C	-1.243969	-1.253447	1.279864
C	-1.162554	2.091075	1.262715
C	-2.567705	1.938259	1.124337
C	-3.181013	2.144591	-0.163247
C	-4.356602	1.296591	-0.581844
C	-4.145713	-0.026174	-0.788885
C	-2.471198	2.836773	-1.128108
C	-1.125894	3.239851	-0.899612

C	-0.465989	2.802773	0.229346
O	0.904856	3.067139	0.347537
C	1.281228	3.984822	1.414331
H	-6.536296	1.094109	2.883787
H	-5.376339	1.702306	4.110237
H	-5.239777	0.014720	3.501230
H	-3.233330	0.875624	4.339061
H	-0.759787	0.567638	4.299983
H	0.451093	0.191194	-1.298646
H	-1.839560	0.232891	-2.253225
H	-3.384146	-1.554449	1.351200
H	-1.087797	-1.623607	2.294309
H	-2.917021	2.991559	-2.111351
H	-0.567200	3.803491	-1.645587
H	2.375180	3.985096	1.448794
H	0.874406	3.657723	2.383533
H	0.912313	4.997213	1.188990
H	2.021262	-0.611743	1.169580
H	1.752607	1.292484	2.719047
H	-5.340985	1.742965	-0.726887
H	-4.965091	-0.679597	-1.095801

References

(1) Fulmer, G. R.; Miller, A. J. M.; Sherden, N. H.; Gottlieb, H. E.; Nudelman, A.; Stoltz, B. M.; Bercaw, J. E.; Goldberg, K. I. NMR Chemical Shifts of Trace Impurities: Common Laboratory Solvents, Organics, and Gases in Deuterated Solvents Relevant to the Organometallic Chemist. *Organometallics* **2010**, *29* (9), 2176-2179. DOI: 10.1021/om100106e.

(2) *Gaussian 16 Rev. C.01*; Wallingford, CT, 2016.

(3) Humphrey, W.; Dalke, A.; Schulten, K. VMD: Visual molecular dynamics. *J. Mol. Graph. Model.* **1996**, *14* (1), 33-38. DOI: 10.1016/0263-7855(96)00018-5.

(4) Hanwell, M. D.; Curtis, D. E.; Lonie, D. C.; Vandermeersch, T.; Zurek, E.; Hutchison, G. R. Avogadro: an advanced semantic chemical editor, visualization, and analysis platform. *J. Cheminform.* **2012**, *4* (1), 17. DOI: 10.1186/1758-2946-4-17.

(5) Yang, R.; Tian, J.; Liu, W.; Wang, Y.; Chen, Z.; Russell, T. P.; Liu, Y. Nonconjugated Self-Doped Polymer Zwitterions as Efficient Interlayers for High Performance Organic Solar Cells. *Chem. Mater.* **2022**, *34* (16), 7293-7301. DOI: 10.1021/acs.chemmater.2c01173.

(6) Rasheed, O. K.; Bailey, P. D.; Lawrence, A.; Quayle, P.; Raftery, J. A Modular Synthesis of Multidentate S-, N- and O-Containing Meta- and Paracyclophanes. *Eur. J. Org. Chem.* **2015**, *2015* (32), 6988-6993. DOI: 10.1002/ejoc.201501058.

(7) Rappe, A. K.; Casewit, C. J.; Colwell, K. S.; Goddard, W. A., III; Skiff, W. M. UFF, a full periodic table force field for molecular mechanics and molecular dynamics simulations. *J. Am. Chem. Soc.* **1992**, *114* (25), 10024-10035. DOI: 10.1021/ja00051a040.

(8) McLean, A. D.; Chandler, G. S. Contracted Gaussian basis sets for molecular calculations. I. Second row atoms, Z=11–18. *J. Chem. Phys.* **1980**, *72* (10), 5639-5648. DOI: 10.1063/1.438980.

(9) Krishnan, R.; Binkley, J. S.; Seeger, R.; Pople, J. A. Self-consistent molecular orbital methods. XX. A basis set for correlated wave functions. *J. Chem. Phys.* **1980**, *72* (1), 650-654. DOI: 10.1063/1.438955.

(10) Peverati, R.; Truhlar, D. G. Improving the Accuracy of Hybrid Meta-GGA Density Functionals by Range Separation. *J. Phys. Chem. Lett.* **2011**, *2* (21), 2810-2817. DOI: 10.1021/jz201170d.

(11) de Meijere, A.; Kozhushkov, S. I.; Rauch, K.; Schill, H.; Verevkin, S. P.; Kümmerlin, M.; Beckhaus, H.-D.; Rüchardt, C.; Yufit, D. S. Heats of Formation of [2.2]Paracyclophane-1-ene and [2.2]Paracyclophane-1,9-diene − An Experimental Study. *J. Am. Chem. Soc.* **2003**, *125* (49), 15110-15113. DOI: 10.1021/ja0374628.

(12) Schleyer, P. v. R.; Williams, J. E.; Blanchard, K. R. Evaluation of strain in hydrocarbons. The strain in adamantane and its origin. *J. Am. Chem. Soc.* **1970**, *92* (8), 2377-2386. DOI: 10.1021/ja00711a030.

(13) Grimme, S.; Mück-Lichtenfeld, C. Accurate Computation of Structures and Strain Energies of Cyclophanes with Modern DFT Methods. *Isr. J. Chem.* **2012**, *52* (1-2), 180-192. DOI: 10.1002/ijch.201100099.

(14) Mann, A.; Hannigan, M. D.; Weck, M. Cyclophanediene and Cyclophanetriene-Based Conjugated Polymers. *Macromol. Chem. Phys.* **2023**, *224* (3), 2200397. DOI: 10.1002/macp.202200397.
